# Involvement of ORAI1/SOCE in Human AML Cell Lines and Primary Cells According to ABCB1 Activity, LSC Compartment and Potential Resistance to Ara-C Exposure

**DOI:** 10.3390/ijms23105555

**Published:** 2022-05-16

**Authors:** Clara Lewuillon, Aurélie Guillemette, Sofia Titah, Faruk Azam Shaik, Nathalie Jouy, Ossama Labiad, Valerio Farfariello, Marie-Océane Laguillaumie, Thierry Idziorek, Adeline Barthélémy, Pauline Peyrouze, Céline Berthon, Mehmet Cagatay Tarhan, Meyling Cheok, Bruno Quesnel, Loïc Lemonnier, Yasmine Touil

**Affiliations:** 1 CNRS, Inserm, CHU Lille, UMR 9020, UMR-S 1277—Canther—Cancer Heterogeneity, Plasticity and Resistance to Therapies, Université de Lille, F-59000 Lille, France; clara.lewuillon@inserm.fr (C.L.); aurelie.guillemette@inserm.fr (A.G.); sofiatitah@gmail.com (S.T.); ossama.labiad@inserm.fr (O.L.); marie-oceane.laguillaumie@inserm.fr (M.-O.L.); thierry.idziorek@inserm.fr (T.I.); ade.barthelemy@gmail.com (A.B.); pauline.peyrouze@inserm.fr (P.P.); celine.berthon@chu-lille.fr (C.B.); meyling.cheok@inserm.fr (M.C.); bruno.quesnel@chu-lille.fr (B.Q.); 2 Institut de Recherche sur le Cancer de Lille (IRCL), F-59000 Lille, France; farukaz29@gmail.com; 3 LIMMS/CNRS-IIS IRL2820, The University of Tokyo, Tokyo 153-8505, Japan; cagatay.tarhan@junia.com; 4 UMS 2014/US41 Plateformes Lilloises En Biologie Et Sante, Université de Lille, F-59000 Lille, France; nathalie.jouy@inserm.fr; 5 Inserm, U1003-PHYCEL-Physiologie Cellulaire, Université de Lille, F-59000 Lille, France; valerio.farfariello@inserm.fr; 6 Laboratory of Excellence, Ion Channels Science and Therapeutics, Université de Lille, F-59655 Villeneuve d’Ascq, France; 7 CNRS, Centrale Lille, Junia, Université Polytechnique Hauts-de-France, UMR 8520—IEMN—Institut d’Electronique de Microélectronique et de Nanotechnologie, Université de Lille, F-59000 Lille, France

**Keywords:** calcium, SOCE, AML, ORAI1, ABCB1, leukemic stem cells, NFAT, Ara-C

## Abstract

Acute myeloid leukemia (AML) is a hematological malignancy with a high risk of relapse. This issue is associated with the development of mechanisms leading to drug resistance that are not yet fully understood. In this context, we previously showed the clinical significance of the ATP binding cassette subfamily B-member 1 (ABCB1) in AML patients, namely its association with stemness markers and an overall worth prognosis. Calcium signaling dysregulations affect numerous cellular functions and are associated with the development of the hallmarks of cancer. However, in AML, calcium-dependent signaling pathways remain poorly investigated. With this study, we show the involvement of the ORAI1 calcium channel in store-operated calcium entry (SOCE), the main calcium entry pathway in non-excitable cells, in two representative human AML cell lines (KG1 and U937) and in primary cells isolated from patients. Moreover, our data suggest that in these models, SOCE varies according to the differentiation status, ABCB1 activity level and leukemic stem cell (LSC) proportion. Finally, we present evidence that ORAI1 expression and SOCE amplitude are modulated during the establishment of an apoptosis resistance phenotype elicited by the chemotherapeutic drug Ara-C. Our results therefore suggest ORAI1/SOCE as potential markers of AML progression and drug resistance apparition.

## 1. Introduction

Acute myeloid leukemia (AML) is a complex hematological malignancy characterized by defective maturation of myeloid primitive cells (blasts). Because AML disease displays genetic, epigenetic and subclonal heterogeneity, it leads to high phenotypic variability, decreasing therapeutic efficiency. Current intensive chemotherapy combining cytarabine (Ara-C) with anthracycline molecules induces complete remission (<5% blasts in the bone marrow) in 70% of patients. Moreover, patients can now benefit (i) from targeted therapies, as well as in cases of unfavorable prognostic factors or even refractory disease, and (ii) from allogeneic hematopoietic stem cell (HSC) transplants or donor lymphocyte transfusion. Despite these tremendous efforts to improve therapeutic efficiency, AML related to relapse leading to death still occurs in more than 50% of cases within 5 years, and these numbers increase up to 80% for subjects over 80 years of age [1]. Mechanisms of anti-leukemia drug resistance that could explain treatment failures have, however, not yet been fully elucidated.

We have previously shown the clinical significance of ATP binding cassette subfamily B-member 1 (ABCB1), also known as permeability glycoprotein and multidrug resistance (P-gp or MDR1), in a cohort of patients with de novo AML [2]. ABCB1 activity and expression were linked to a worse prognosis and leukemic stem cell (LSC) compartment proportion. However, we showed that ABCB1 does not directly mediate chemotherapy resistance and can represent a bystander effect related to other chemoresistance mechanisms [2]. Deciphering these mechanisms related to the ABCB1 phenotype and underlying chemoresistance in AML is crucial to better eradicate disease relapse, which is particularly frequent in this hematological malignancy.

Dysregulation in calcium signaling and/or homeostasis affects numerous cellular functions and has been shown to be involved in cancer initiation and progression. Altered calcium signaling can lead to cancer development via its impact on various processes, including gene transcription, regulation of cell cycle engagement, proliferation, differentiation and apoptosis, all of which potentially contribute to the development of resistance to cancer therapies [3].

One of the main calcium entry pathways into cells is store-operated Ca^2+^ entry (SOCE), also known as capacitative calcium entry, mediated by store-operated channels (SOCs). SOCE involves the activation of plasma membrane ORAI calcium channels [4]. We and others have previously shown that ORAI1 mediates SOCE in cancer cell lines and plays a role in proliferation and chemoresistance [5,6,7].

Alterations in calcium signaling have been extensively studied in solid cancers [3]. In hematological malignancies such as myeloid leukemia, few studies have reported data regarding dysregulation in calcium signaling pathways and the associated cellular functions [8]. In AML, the roles of ORAI calcium channels have rarely been investigated [8], in contrast to the calcium/calcineurin/Nuclear factor of Activated T cells (NFAT) pathway, one of the main signaling pathways activated by calcium [9]. It has been reported that primary AML cells exhibit variable NFAT expression according to the stage of differentiation and disease progression [9]. Indeed, NFAT was shown to be overexpressed in primary leukemic blasts at disease relapse compared with diagnosis [10]. Interestingly, inhibition of NFAT nuclear translocation increased the sensitivity of AML cells to chemotherapy drugs from patients bearing the internal tandem duplication of the FLT3 receptor (FLT3ITD), a frequent and disease driver mutation, thus emphasizing a potential link between calcium signaling and chemoresistance [11].

Here, we demonstrate for the first time, to our knowledge, the involvement of ORAI1 in SOCE in human AML cell lines and primary cells according to their differentiation status, ABCB1 activity and LSC compartment proportion. We also show the contribution of ORAI1 (i) to AML cell cycle engagement and (ii) to apoptosis resistance against Ara-C treatment.

## 2. Results

### 2.1. ORAI1 and ABCB1 Gene Expression Varies in AML Patients at Diagnosis According to the Differentiation Stage and Prognosis of the Disease

#### 2.1.1. ORAI1 and ABCB1 Are Overexpressed in Peripheral Blood Mononuclear Cells (PBMCs) Isolated from AML Patients

**Figure 1:**
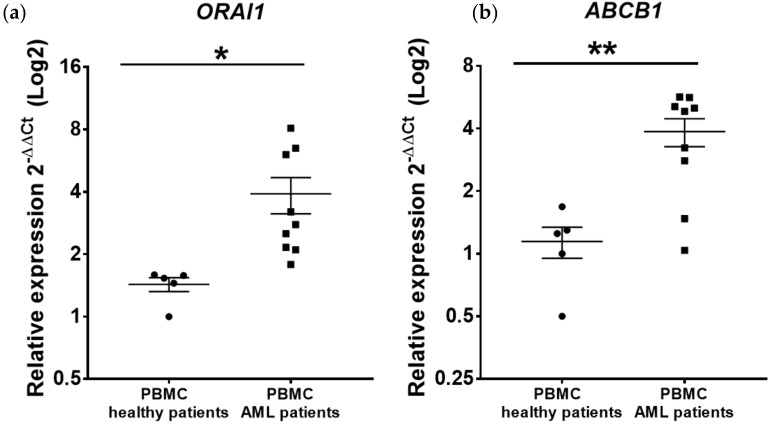
Expression of *ORAI1* and *ABCB1* in human AML primary cells compared with healthy primary PBMCs. qRT–PCR detection of *ORAI1* (**a**) and *ABCB1* (**b**) gene expression in AML primary cells isolated from 9 patients at disease diagnosis compared with normal primary PBMCs isolated from 5 healthy donors. The expression levels of *ORAI1* and *ABCB1* relative to beta-2 microglobulin (*B2M*) for each sample were normalized to the *ORAI1* and *ABCB1* levels in healthy PBMCs. The relative expression ratio for each gene was calculated by the 2^−ΔΔCt^ method. * *p* < 0.05, ** *p* < 0.01 compared with healthy PBMCs.

We first analyzed *ORAI1* and *ABCB1* gene expression by RT–qPCR in PBMCs isolated from nine AML patients (Table S1) at the time of initial diagnosis (>80% of leukemic cells) compared with PBMCs from healthy donors. We found that *ORAI1* gene expression was increased in leukemic cells compared with normal cells (normal PBMCs 2^−∆∆Ct^ = 1.4 vs. 3.91 for leukemic cells, *p* = 0.032) (Figure 1a). In the same manner, *ABCB1* gene expression was also significantly increased in leukemic blasts compared with normal PBMCs (normal PBMCs 2^−∆∆Ct^ = 1.14 vs. 3.86 for leukemic cells, *p* = 0.0063) (Figure 1b).

#### 2.1.2. ORAI1 and ABCB1 Gene Expression Varies with the Stage of AML Differentiation and Prognostic Classification

**Figure 2:**
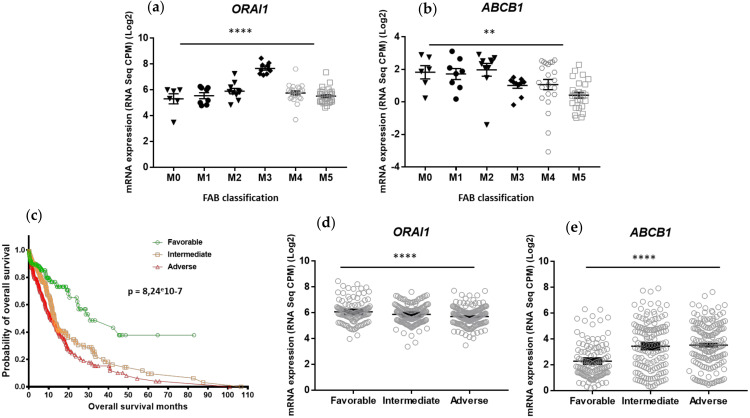
Differential expression patterns of *ORAI1* and *ABCB1* in AML primary cells isolated from patients at the time of disease diagnosis, according to the FAB classification and ELN 2017 risk stratification. (**a**–**e**) RNA-seq data were generated by Tyner et al. [12] from samples derived from patients with AML at disease diagnosis. RNA-Seq data were obtained from public domain AML datasets (https://www.cbioportal.org, accessed on 15 March 2022). Data were extracted as described by [13,14] for *ORAI1* and *ABCB1* expression in AML primary cells according to the FAB classification (**a**,**b**). Overall survival of 439 adult patients with de novo AML according to ELN2017 risk stratification (favorable, intermediate, adverse). The *p* value is based on the log rank test. (**c**). Data were extracted for *ORAI1* (**d**) and *ABCB1* (**e**) expression according to ELN2017 risk stratification. Normalized expression values are presented as CPM; *p* values are from one-way ANOVA tests comparing the different means (** *p* < 0.01; **** *p* < 0.0001).

In addition to the fresh AML primary samples collected by our laboratory, we analyzed RNA-seq datasets from a cohort of human AML samples at diagnosis (*n* = 439, [12]) available in the public domain (https://www.cbioportal.org) for *ORAI1* and *ABCB1* gene expression. We first compared AML patient samples according to the stage of leukemic cell differentiation (French–American–British (FAB) classification), i.e., from the M0 undifferentiated stage to the M5 monocytic differentiated stage. We observed an increase in *ORAI1* expression associated with late stages of AML differentiation (one-way ANOVA (F(5.79) = 18.07, *p* < 0.0001) (Figure 2a), while in an opposite manner, *ABCB1* expression was downregulated in a more advanced stage of differentiation (one-way ANOVA (F(5.76) = 4.2, *p* = 0.002) (Figure 2b).

AML patients were then classified as favorable (*n* =117), intermediate (*n* = 143) and adverse risk (*n* = 157) based on the 2017 European Leukemia Net (ELN) risk stratification (Figure 2c). We analyzed ORAI1 and ABCB1 gene expression in these three ELN classes, and we observed a significant increase in *ABCB1* in AML patient samples with adverse risk (one-way ANOVA (F(2.414) = 21.16, *p* < 0.0001)), while *ORAI1* gene expression was downregulated in this ELN class (one-way ANOVA (F(2.414) = 7.547, *p* = 0.0006)) (Figure 2d,e).

#### 2.1.3. AML with High ABCB1 and Low ORAI1 Gene Expression Shows Reduced Expression of CD33 (Differentiation Marker) and CDK4/CDK6 (G0–G1 Transition Phase Regulators)

**Figure 3:**
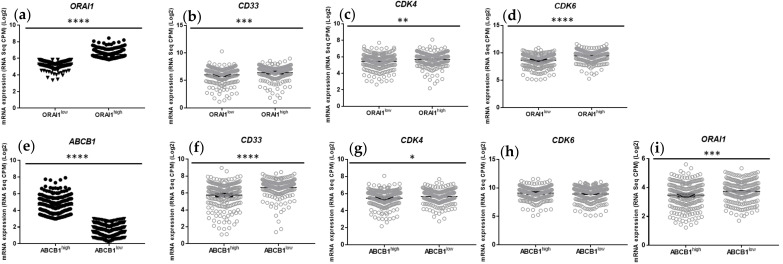
Expression of *CD33, CDK4* and *CDK6* in primary AML cells isolated from patients at the time of diagnosis, according to *ABCB1* and *ORAI* gene expression. (**a**–**i**) RNA-seq data were generated by Tyner et al. [12] from samples derived from patients with AML. RNA-Seq data were obtained from public domain AML datasets (https://www.cbioportal.org). Data were extracted as described in [13,14] for *ORAI1* and *ABCB1* expression, and 2 groups (*n* = 220 and *n* = 219) of AML patients were discriminated according to high or low levels of *ORAI1* (**a**) and *ABCB1* (**e**) expression. Data were extracted for *CD33, CDK4* and *CDK6* gene expression in the *ORAI1^low^* and *ORAI1^high^* AML and in the *ABCB1^low^* and *ABCB1^high^* AML patient subgroups. Normalized expression values are presented as CPM; *p* values were obtained from Student’s t tests between means (* *p* < 0.05; ** *p* < 0.01; *** *p* < 0.001; **** *p* < 0.0001).

We next analyzed the expression of genes related to AML differentiation (*CD33*) and to cell cycle regulation, more specifically to G0–G1 transition phase regulators (*CDK4* and *CDK6*), in AML patient samples discriminated by their level of expression of *ORAI1* (*ORAI1^low^* and *ORAI1^high^*, *n* = 219 and *n* = 220, respectively) (Figure 3a) or *ABCB1* (*ABCB1^low^* and *ABCB1^high^*, *n* = 220 and *n* = 219, respectively) (Figure 3e) genes. We found that both *ABCB1^high^* and *ORAI1^low^* AML were associated with lower expression of *CD33* (Log (2) = 6.661 vs. 5.761, *p* < 0.0001 and Log (2) = 6.436 vs. 6.003, *p* = 0.0007, respectively) and *CDK4* (Log (2) = 5.658 vs. 5.464, *p* = 0.0211 and Log (2) = 5.698 vs. 5.424, *p* = 0.0011, respectively) genes (Figure 3). Regarding the *CDK6* gene, only *ORAI1^low^* AML displayed lower expression than *ORAI1^high^* AML (Figure 3d,h). Interestingly, *ABCB1^high^* AML samples displayed an overexpression of several leukemic stem cell markers previously identified and established as the 17-gene stemness signature [15] (Table S2) and underexpression of the *ORAI1* calcium channel gene (Log (2) = 3.471 vs. 3.741, *p* < 0.001) (Figure 3i).

Collectively, these data point out a possible link between the high gene expression of *ABCB1* and the low expression of *ORAI1* with (i) an undifferentiated stage of AML, (ii) lower expression of G0–G1 transition phase regulators, (iii) higher expression of genes related to the leukemic stem cell phenotype and (iv) an overall worse prognosis for patients.

### 2.2. Involvement of ORAI1 in SOCE in AML Cell Lines According to ABCB1 Activity, Stem Cell Phenotype and Cell Cycle Engagement

#### 2.2.1. ABCB1 Activity Reflects Stemness Markers and Cell Cycle Engagement in AML Cells

We have previously shown in numerous cohorts of AML patients that *ABCB1* gene expression is strongly linked to ABCB1 activity, the LSC proportion and a worse disease prognosis [2]. Consequently, we next questioned the role of ORAI1 in SOCE in leukemic cells with higher ABCB1 activity and/or an enrichment in stemness markers.

Therefore, we investigated whether ORAI1 contributes to SOCE in two representative AML human cell lines, KG1 and U937, which display opposite features regarding (i) stage of differentiation, (ii) ABCB1 expression and (iii) LSC size compartment [16,17]. We first analyzed the CD34 + CD38− (i.e., LSC) subpopulation in the KG1 and U937 AML cell lines by qRT–PCR, LSC-associated and ABCB1 gene expression and flow cytometry. As previously reported, the immature KG1 acute myeloid leukemia cell line overexpressed LSC-associated and *ABCB1* genes and made up an important CD34 + CD38− LSC subpopulation (10.4%). In contrast, the U937 monocytic differentiated leukemia cell line exhibited a downregulation of LSC markers and a decrease in *ABCB1* gene expression (Table 1).

**Figure 4:**
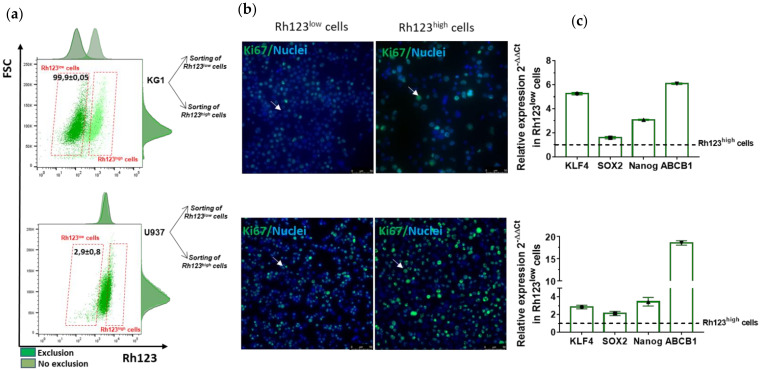
Flow cytometry identification of Rh123^low^ (ABCB1^high^) populations enriched with quiescent cells and stem cell markers in KG1 and U937 AML cell lines. (**a**) KG1 and U937 leukemic cells were incubated with 0.1 μg/mL Rh123 probe for 20 min. After 60 min of exclusion of the Rh123 probe, the Rh123^low^ cell subpopulation appeared/is highlighted. (**b**) Ki67 (Alexa Fluor 488, green) and nuclear (Hoechst, blue) staining was then performed on cells sorted by FACS based on Rh123 probe exclusion. Ki67-negative or Ki67-low-expressing cells were considered quiescent cells or slow-cycling cells, respectively. White arrows indicate quiescent cells in the Rh123^low^ compartment and cycling cells in the Rh123^high^ cell compartment. (**c**) Stemness-associated gene expression was analyzed by RT–qPCR and is shown in the Rh123^low^ cells relative to the expression of the corresponding genes in Rh123^high^ cells. The relative expression ratio for each gene was calculated by the 2^−ΔΔCt^ method. The calculated values represent the expression level of each gene relative to the expression of *B2M* used herein as an endogenous control. Histograms present the mean results from three independent experiments.

We next evaluated ABCB1 activity in both AML cell lines using the functional Rhodamine 123 (Rh123) exclusion assay. The exclusion rate of the Rh123 fluorescent probe was assessed by flow cytometry. With this method, we characterized two distinct cell populations among U937 and KG1 cells (Figure 4a). Cells displaying a high fluorescence intensity (i.e., cells that did not exclude the Rh123 probe) were considered Rh123^high^ cells, reflecting lower ABCB1 activity. Cells with a lower fluorescence intensity, noted Rh123^low^, excluded the probe, reflecting higher ABCB1 activity. A discrete population of Rh123^low^ cells was identified among the differentiated U937 cell line (2.9 ± 0.8%), while a majority of the immature KG1 cells were characterized as Rh123^low^ (99.9 ±0.05%) (Figure 4a).

We and others have previously identified an enrichment of stem-like characteristics (e.g., quiescence and overexpression of stemness genes) in normal hematopoietic cells, tumor cell lines and primary cancer cells using the Rhodamine (Rh123) functional exclusion assay [18,19].

Rh123^low^ and Rh123^high^ U937 and KG1 sorted cells were then subjected to immunostaining and qPCR. Immunocytochemistry revealed a significant difference in the expression level of the nuclear protein Ki67, which is expressed during the cell cycle, between the Rh123^low^ and Rh123^high^ populations in both leukemia cell lines (Figure 4b). The absence or the low fluorescence intensity of the Ki67 protein observed in Rh123^low^ cells was associated with quiescence or slow cycling, respectively. An enrichment of stemness characteristics within KG1 and U937 Rh123^low^ cells was further confirmed by qPCR analysis, which showed a higher expression level of the stemness genes *KFL4*, *SOX2* and *NANOG* (Figure 4c). As expected, *ABCB1* gene expression was highly increased in KG1 and U937 Rh123^low^ cells (Figure 4c).

In addition, we performed flow cytometry immunophenotyping of the Rh123^low^ and Rh123^high^ populations in both cell lines with regard to CD34 and CD38 surface marker expression (Table 2). We observed an enrichment of the CD34 + CD38− stem-cell-like phenotype in the Rh123^low^ cell compartment (a 34-fold and 5-fold increase in U937 and KG1 cells, respectively).

Collectively, these results showed that the Rh123^low^ cell compartment in both cell lines reflected common characteristics regarding ABCB1 activity or the LSC phenotype. Importantly, despite all the common features between the Rh123^low^ compartment, we observed a marked difference between the proportions of quiescent Ki67-negative cells, which were more pronounced in the U937 Rh123^low^ population, while the number of slow-cycling cells (Ki67^low^) was increased in KG1 Rh123^low^ cells (Figure 4).

#### 2.2.2. The Involvement of ORAI1 in SOCE in AML Cell Lines Depends on ABCB1 Activity and LSC Status

We first assessed the contribution of ORAI1 to SOCE in the U937 and KG1 leukemia cell lines. Both cell lines were either treated with Synta66 (SOC inhibitor) or transfected with siORAI1 (100 nM, 48 h) or siCTL and were then loaded with the fluorescent ratiometric Indo-AM calcium dye. The 13 min kinetics were monitored by flow cytometry, during which thapsigargin (1 µM), an inhibitor of SERCA pumps, was added after 2 min in the absence of extracellular calcium followed by the addition of extracellular Ca^2+^ (2 mM) after 9 min. This protocol with thapsigargin induces depletion of the endoplasmic reticulum (ER) calcium stores and activation of SOCE visualized at the time when calcium is added to the extracellular calcium. As shown in Figures S1 and S2, we monitored over time the ratio of F400/F475 fluorescence intensity reflecting cytosolic calcium levels in both cell lines. Application of the SOC channel inhibitor Synta66 (10 µM) and *ORAI1* silencing in KG1 and U937 cells elicited a marked decrease in SOCE (Figure S1 and S2), thus confirming the involvement of the ORAI1 channel in SOCE in AML cell lines.

**Figure 5:**
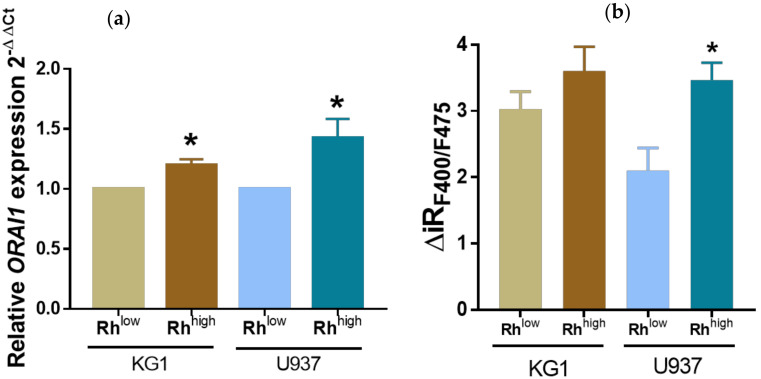
SOCE is downregulated in the KG1 and U937 Rh123^low^ cell subpopulations. (**a**) Relative *ORAI1* expression in KG1 and U937 Rh123^low^ and Rh123^high^ cell subpopulations determined by RT–qPCR. *ORAI1* relative expression was calculated by the 2^−ΔΔCt^ method. * *p* < 0.05. (**b**) [Ca^2+^]_i_ was monitored by flow cytometry, and the F400/F475 ratio reflected calcium capacitive entry in KG1 and U937 Rh123^low^ and Rh123^high^ cell subpopulations. The graphs represent the means of 3 independent experiments. * *p* ≤ 0.05.

We next evaluated the level of expression of the ORAI1 calcium channel in the sorted Rh123^low^ and Rh123^high^ populations. As shown in Figure 5a,b, KG1 and U937 Rh123^low^ cells, which displayed high ABCB1 expression and activity, exhibited lower levels of ORAI1 expression and activity.

The advantage of the multiparameter flow cytometry technique is the possibility of performing a functional assay of rhodamine exclusion while simultaneously monitoring intracellular calcium levels without unnecessary additional sorting experiments. As shown in Figure 5b, we observed a slight decrease in SOCE in KG1 and U937 Rh123^low^ cells compared with Rh123^high^ cells. Application of the SOC channel inhibitor Synta66 (10 µM) led to a dramatic decrease in SOCE amplitude in KG1 and U937 Rh123^low^ and Rh123^high^ compartments. Interestingly, the inhibition elicited by Synta66 was similar in both compartments, indicating similar SOCE properties in the Rh123^low^ and Rh123^high^ populations (Figure S3).

#### 2.2.3. ORAI Is Involved in AML Cell Line Proliferation and Cell Cycle Engagement

**Figure 6:**
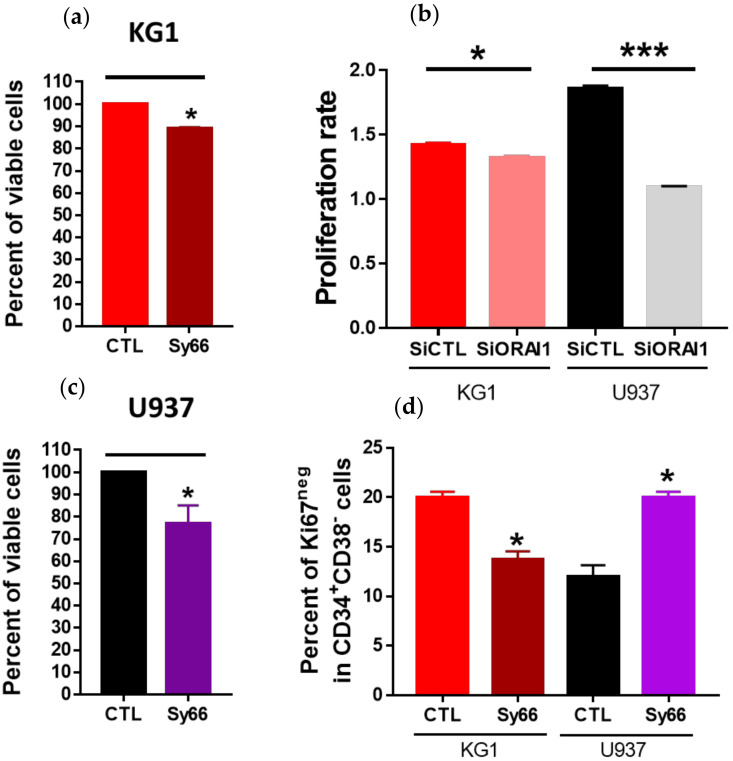
SOCs/ORAI1 are involved in proliferation of KG1 and U937 cells and in the quiescence state of LSC. KG1 (**a**) and U937 (**c**) viability (Trypan blue) under control conditions or after 24 h of treatment with synta66 (10 µM). (**b**) Proliferation rate of KG1 and U937 cells transfected with control or ORAI1 siRNA. (**d**) Percentage of Ki67^neg^ cells in CD34 + CD38− LSCs treated or not treated for 24 h with synta66 (10 µM). For each experiment, three independent experiments were performed. *** *p* ≤ 0.001. * *p* ≤ 0.05.

While it has been described that SOC inhibitors can induce proliferation defects in cancer cell lines [20], the involvement of SOC channels in AML cell proliferation remains largely unknown [8]. However, the role of [Ca^2+^]_i_ and associated signaling pathways in cell cycle engagement and/or proliferation of AML primary cells and cell lines has been investigated [8]. Using the SOC channel inhibitor Synta66, we observed a small but significant decrease in KG1 and U937 proliferation after a 24 h treatment compared with control cells (Figure 6a,b) without any significant cell death engagement. Similar to Synta66 application, siRNA against ORAI1 also induced a decrease in proliferation in both AML cell lines (Figure 6c).

SOC inhibition by Synta66 or with the abolition of *ORAI1* expression (siRNA) did not induce any significant change in ABCB1 activity (% of Rh123^low^ cells) or the proportion of CD34 + CD38− leukemic stem cells in either AML cell line (Table S3). We then stained the cells for Ki67/PI simultaneously with CD34 and CD38 surface markers to check cell cycle engagement, particularly the size of the LSC compartment, after treatment with Synta66. Interestingly, we observed a significant increase in the proportion of quiescent CD34 + CD38− leukemic stem cells in the U937 cell line (Figure 6d). In the CD34 + CD38− KG1 cell compartment, the number of Ki67-negative cells was decreased (Figure 6d) due to an accumulation in the G1 phase of the cell cycle (data not shown).

### 2.3. Cytarabine (Ara-C) Has an Impact on SOCE in AML Cell Lines and Primary Cells Depending on ABCB1 Activity, the LSC Proportion and/or Cell Cycle Engagement

We next evaluated the impact of cytarabine (Ara-C), a chemotherapeutic agent currently used in the treatment of AML, on KG1 and U937 cell lines and primary cells to determine the role and contribution of ABCB1 activity or LSC proportion in the response to this drug.

#### 2.3.1. Effect of Ara-C on the AML Cell Cycle and LSC Proportion

**Figure 7:**
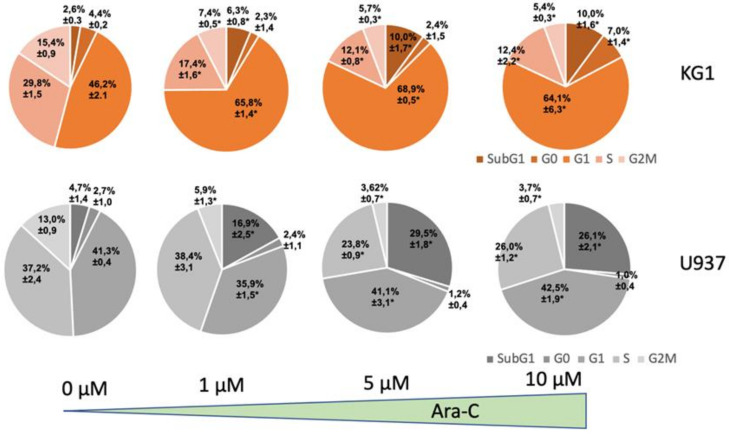
Effect of Ara-C on the KG1 and U937 cell cycle. Cell cycle analysis by flow cytometry of KG1 and U937 AML cell lines with KI67/PI double staining. Cells were either in a control condition or treated with Ara-C at the indicated concentrations for 24 h. Three independent experiments were performed. * *p* ≤ 0.05.

As expected, a 24 h treatment with Ara-C inhibited U937 and KG1 cell proliferation in a dose-dependent manner. U937 cells were more sensitive to the treatment than KG1 cells, with the latter displaying higher ABCB1 activity and a higher proportion of LSCs (Figure S4). We also performed a cell cycle analysis by flow cytometry (Ki67/PI double staining) and observed that KG1 cells accumulated in the G1 phase (46.2% ± 2.1 vs. 65.8% ±1.4, CTL and 1 µM Ara-C, respectively) with a slight increase in cell death (2.6% ± 0.3 vs. 6.3% ± 0.8 of cells in subG1 with 1 µM Ara-C) (Figure 7 and Figure S5). U937 cells showed a more significant increase in cell death than KG1 cells after cytarabine treatment (16.9% ± 2.5 of cells in subG1 at 1 µM Ara-C compared to 6.3% ± 0.8 for U937 and KG1, respectively). Moreover, we did not observe any change in the percentage of cells in the G0 phase of the cycle after treatment with Ara-C for either cell line (Figure 7 and Figure S5).

Ara-C treatment increased the Rh123^low^ cells and decreased the Rh123^high^ cell compartments in a dose-dependent manner for U937 cells but not for KG1 cells (Table S5). However, the proportion of CD34+ CD38− KG1 cells increased in a dose-dependent manner with this treatment (Table S5). As expected, our cell cycle results (% of cells in SubG1) showed that cells undergoing cell death were cells with low ABCB1 activity (Rh123^high^), while CD34+ CD38− and Rh123^low^ cells were more resistant to Ara-C.

#### 2.3.2. Effect of Ara-C on SOCE in AML Leukemic Cells

##### The Effect of Ara-C on SOCE in AML Cell Lines Depends on ABCB1 Activity and the LSC Phenotype

Considering the difference in chemosensitivity and LSC compartment size between KG1 and U937 cells and the potential role of SOC activity in these features, our next goal was to functionally study the impact of Ara-C on SOCE in both cell lines according to ABCB1 activity. To assess SOCE in viable cells only, we measured SOCE by flow cytometry in cells treated or not with Ara-C and stained with a viability dye.

**Figure 8:**
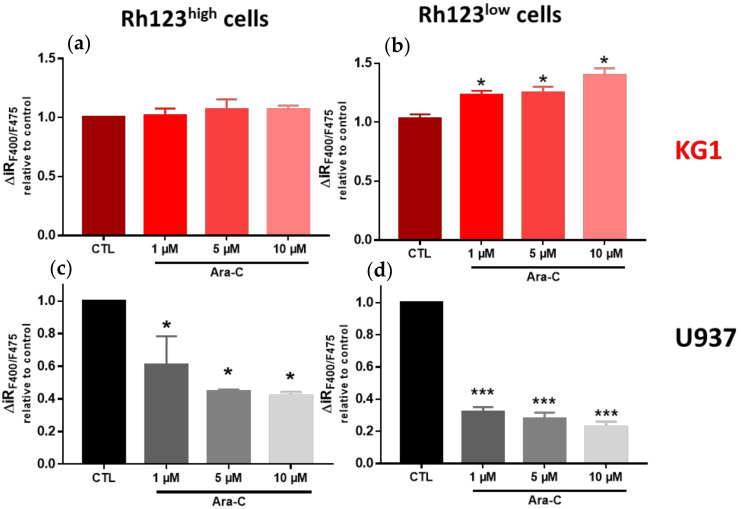
Effect of Ara-C on SOCE in Rh123^high^ and Rh123^low^ KG1 and U937 cell subpopulations. Cells were treated for 24 h with Ara-C at the indicated concentrations. For each condition, three independent experiments were performed. Flow cytometric analysis of the impact of Ara-C on SOC channel activity in the Rh123^high^ (**a**,**c**) and Rh123^low^ (**b**,**d**) KG1 and U937 cell subpopulations. The delta of the F400/F475 ratio reflects the capacitative Ca^2+^ entry in the Rh123^high^ and Rh123^low^ cell subpopulations. * *p* ≤ 0.05; *** *p* < 0.001.

In the more chemoresistant KG1 Rh123^low^ cells (high ABCB1 activity), Ara-C exposure induced a slight increase in capacitative calcium entry in a dose-dependent manner, without any significant impact on the basal calcium level (Figure 8 and Figure S6). In contrast, the more chemosensitive U937 Rh123^high^ cells (low ABCB1 activity) displayed a marked decrease in SOCE with a slight increase in basal calcium levels after Ara-C treatment. Increased SOC activity could therefore be linked to resistance mechanisms mobilized by AML leukemic cells. Surprisingly, when we assessed SOC activity in the minority cell compartment among KG1 and U937 cells regarding the ABCB1 activity level (i.e., KG1 Rh123^high^ and U937 Rh123^low^ cells), these cells displayed either no change (KG1) or a decrease (U937) in SOCE following Ara-C treatment (Figure 8 and Figure S6).

**Figure 9:**
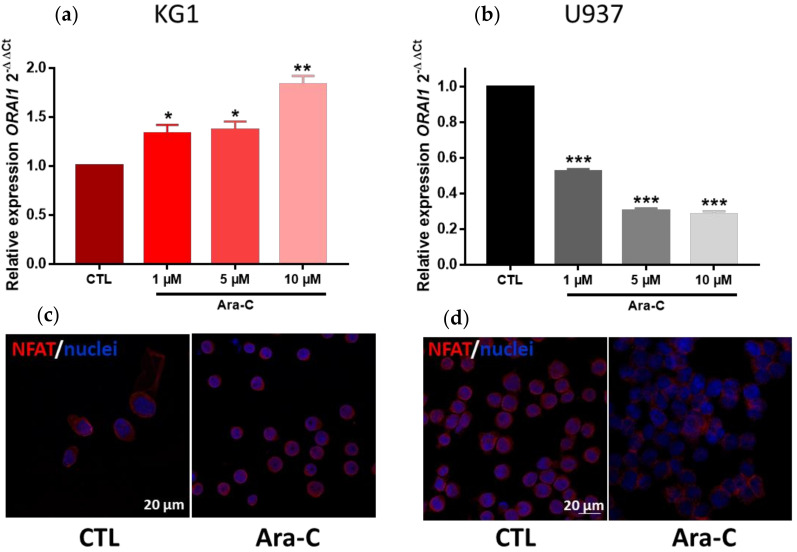
Effect of Ara-C on *ORAI1* expression and NFAT localization. Relative expression of the calcium channel *ORAI1* in KG1 (**a**) and U937 (**b**) leukemic cell lines. *ORAI1* expression was analyzed by RT–qPCR. The relative expression ratio for ORAI1 was calculated by the 2^−ΔΔCt^ method. * *p* < 0.05; ** *p* < 0.01; *** *p* < 0.001. Confocal microscopy showing NFAT expression and localization (Alexa Fluor 488, red) and nuclei (Hoechst, blue) in control and Ara-C-treated (5 µM) KG1 (**c**) or U937 (**d**) cells. The scale bar is indicated.

We next evaluated *ORAI1* gene expression in KG1 and U937 cells following Ara-C exposure. We observed a significant increase in *ORAI1* expression in KG1 cells and a significant decrease in U937 cells (Figure 9), in agreement with the observed Ara-C functional effects on SOCE in both leukemia cell lines.

Because the calcineurin–NFAT signaling pathway is closely associated with SOCE and ORAI channels in several models, we next evaluated NFAT localization in both KG1 and U937 AML cell lines treated or not treated with Ara-C for 24 h. Under control conditions, NFAT was predominantly localized in KG1 cytoplasm and in U937 nuclei, as shown in Figure 9. After Ara-C exposure, KG1 exhibited a translocation of NFAT to the nucleus (Figure 9). Conversely, U937 cells treated with Ara-C displayed a predominant cytoplasmic distribution of NFAT (Figure 9).

##### Ara-C Affects SOCE in Primary AML Cells from Patients According to ABCB1 Activity and LSC Phenotype

We first performed a Rh123 exclusion assay and CD34 + CD38− immunophenotyping in AML primary cells from nine patients (Table S1) to screen their ABCB1 activity and LSC compartment proportion. We next evaluated the Ara-C effect on high ABCB1 activity and LSC-enriched AML primary cells (AML patient #1) compared with lower ABCB1 activity and poorly LSC-enriched AML cells (AML patient #2) (Table S6).

As expected, Ara-C exposure induced a slight decrease in AML#1 primary cell viability and a more pronounced decrease in AML#2 cells (Figure S7). Similarly, cell death was significantly higher in AML#2 Ara-C-treated cells than in AML#1 cells (48 vs. 20% of subG1, respectively) (Figure S7).

**Figure 10:**
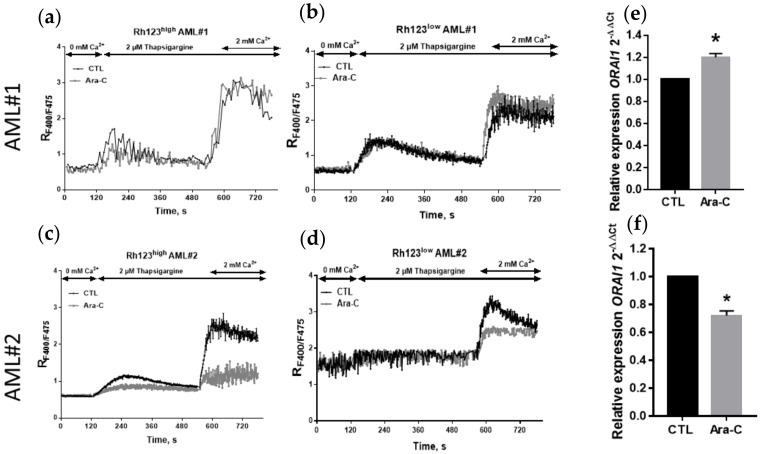
Effect of Ara-C on SOCE and *ORAI1* expression in AML#1 and AML#2 primary cells according to ABCB1 activity. Cells were treated or not for 24 h with 5 µM Ara-C. Three independent experiments were performed. Time course of [Ca^2+^]_i_ analyzed by flow cytometry (Indo-AM) upon SOC activation in AML#1 and AML#2 Rh123^high^ (**a**,**d**,**c**) and Rh123^low^ (**b**,**d**) primary cells. Each point represents the mean of 3 independent experiments including between 1000 and 10,000 cells. Relative expression of *ORAI1* in AML patient #1 and #2 cells (**e**,**f**). *ORAI1* expression was analyzed by RT–qPCR. The relative expression ratio was calculated by the 2^−ΔΔCt^ method. * *p* < 0.05.

In the more chemoresistant and LSC-enriched AML#1 Rh123^low^ cells (high ABCB1 activity), Ara-C treatment provoked a slight increase in capacitive calcium entry (Figure 10). Conversely, the more chemosensitive AML#2 cells (low ABCB1 activity) displayed a significant decrease in SOCE subsequent to Ara-C treatment (Figure 10).

We then evaluated *ORAI1* gene expression in AML#1 and AML#2 primary cells following Ara-C exposure, and we observed, in agreement with the Ara-C effect on SOCE, a significant increase in AML#1 cells with high ABCB1 activity and a significant decrease in AML#2 primary cells (Figure 10).

Interestingly, we observed similar SOCE and ORAI1 expression modulation in primary cells and KG1/U937 cell lines according to their respective ABCB1 activity, LSC status and differentiation stage.

To verify NFAT localization, we performed immunocytochemistry staining, and we observed results similar to those obtained with KG1 and U937 cell lines, namely, a predominant redistribution of NFAT in the nucleus of AML#1 cells and in the cytoplasm of AML#2 cells after Ara-C treatment, as shown in Figure S8.

## 3. Discussion

In this study, we present new data supporting that ORAI1 calcium channels mediate SOCE in human AML cell lines and primary cells from patients. We show that ORAI1/SOCE plays a role in AML cell proliferation and in G0–G1 cell cycle engagement according to the LSC phenotype. We also demonstrate a link between ORAI1 expression and/or activity and AML cell differentiation stage, ABCB1 activity and the LSC compartment. Furthermore, our data suggest that Ara-C treatment modulates SOCE in AML cell lines and primary cells via ORAI1 expression regulation. Using KG1 and U937 AML cell lines, two models with distinct ABCB1 activity and LSC compartment proportions, we observed opposite responses to Ara-C treatment with regard to ORAI1 expression and SOCE modulation. The more chemoresistant ABCB1^high^ and LSC-enriched KG1 AML cells displayed an increase in SOCE and ORAI1 expression following Ara-C treatment, while the chemosensitive ABCB1^low^ and poorly LSC-enriched U937 cells exhibited the opposite responses. Interestingly, we observed similar behaviors in primary AML cells obtained from patients at the time of diagnosis and presenting the same opposite features.

We have previously shown the clinical significance of ABCB1 in a cohort of patients with *de novo* AML and highlighted the link between ABCB1 expression and activity with LSC gene expression and risk stratification [2]. However, ABCB1 was not directly linked to drug resistance, prompting us to propose a bystander effect of these particular MDRs in chemoresistance mechanisms. Interestingly, in this study, we present new data obtained from public datasets [12], corresponding to the RNA-Seq analyses of a cohort of 439 AML patients at diagnosis, linking high ABCB1 expression and low ORAI1 expression with (i) an undifferentiated stage of AML, (ii) a lower expression of CDK4 and CDK6 (i.e., G0–G1 transition phase regulators), (iii) a higher expression of genes related to the LSC phenotype and (iv) an overall worse prognosis of the disease. In addition, we analyzed the genetic phenotypes associated to the different ABCB1 and ORAI1 expression levels. We observed in the ABCB1^high^ group a significant increase in AML subgroups bearing RUNX1, TP53 and GATA2 mutations, and a decrease in AML patients presenting NPM1 and FLT3-ITD mutations. In the ORAI1^low^ group, NPM1 and FLT3-ITD mutations were seldom observed, while AML bearing the RUNX1 mutation was over-represented. These observations were in agreement with ELN 2017 risk stratification: FLT3-ITD and NPM1 mutations are associated with good prognosis, while TP53, GATA2 and RUNX1 are correlated with an adverse risk.

We functionally confirmed these observations not only in the two representative AML cell lines KG1 and U937 but also in primary cells (AML#1 and AML#2) isolated from leukemia patients at the time of diagnosis based on their expression levels of ABCB1 and LSC-associated markers. In agreement with our results obtained by screening a public dataset, the slow-cycling immature KG1 AML and AML#1 primary cells displayed high ABCB1 activity and an enrichment of their LSC compartment while underexpressing ORAI1. In contrast, fast-cycling differentiated U937 and AML#2 primary cells exhibited lower ABCB1 activity and a discrete number of LSCs while overexpressing ORAI1.

More precisely, we identified two distinct cell subpopulations among KG1 and U937 cells in regard to ABCB1 activity, namely, the Rh123^low^ and Rh123^high^ populations. The Rh123^low^ cell compartment in each cell line shared common characteristics regarding ABCB1 activity and the LSC phenotype characterized herein by expression markers (e.g., *SOX2, KLF4, NANOG*), surface markers (CD34 + CD38-) and Ki67 expression levels. Interestingly, we observed that ORAI1 expression was downregulated in Rh123^low^ cells compared with their Rh123^high^ counterparts. Emerging roles of Ca^2+^ in cancer stem cell (CSC) population maintenance and stemness have been reported, and some studies have proposed a link between ORAI1 and the regulation of the CSC compartment [21]. For instance, it has been shown that ORAI1 and SOCE, through NFAT activation, promote stemness in oral/oropharyngeal squamous cell carcinoma. Specifically, the ALDH^high^ CSC population expressed more ORAI1 proteins, and a study showed that ectopic expression of ORAI1 in nontumorigenic immortalized oral epithelial cells resulted in increased proliferation, self-renewal, and tumor-initiating capacities [22]. These data therefore contradict our observations in the Rh123^low^ population, which was enriched in LSCs and expressed less ORAI1. However, similar to our results, it has been reported that SOCE inhibition triggers glioblastoma stem cells to adopt a quiescent state, suggesting that the transition from proliferation to quiescence involves the remodeling of Ca^2+^ signaling [23]. In our study, while the proportion of LSCs was not altered by SOCE modulation through ORAI1 silencing, LSCs were more engaged in quiescence in U937 CD34 + CD38− LSC stem cells. In hematopoietic stem cells (HSCs), two elegant studies on how intracellular calcium levels dictate cell fate (i.e., quiescence or cycling state) have shown opposite results [8]. Bonora et al. demonstrated an increase in the intracellular Ca^2+^ concentration during the switch from the G0 to G1 state and that cycling HSCs display active NFAT and TET2 degradation [24]. Conversely, Fukushima et al. proposed that the intracellular Ca^2+^ concentration decreases during the G0 to G1 transition, leading to an increase in CDK4/6 activity, and that the calmodulin (CaM)/CaM kinase (CaMK) pathway is involved in HSC quiescence [25]. These apparently contradictory results could be explained by the difficulty in discriminating between different stages of “activation” of normal and cancer stem cells. In addition, symmetric and asymmetric CSC divisions could also increase the level of complexity to understand the regulation of CSCs/LSCs by calcium signaling. Combined with the heterogeneity of responses observed in different organs and tissues, these reports emphasize the need to better understand calcium remodeling during the transition between the different activation stages of LSCs.

While alterations in calcium signaling have been extensively studied in solid cancers, only a few studies have explored the dysregulation of calcium-dependent signaling pathways in AML, and none have investigated calcium-dependent resistance mechanisms [8]. Regarding the molecular nature of SOCE in AML, to our knowledge, only one study has proposed that ORAI1 and ORAI2 mediate SOCE in the HL60 AML cell line while demonstrating their role in proliferation and migration [26]. In the present study, we are the first group to provide evidence of the involvement of ORAI1 in AML cell lines and in primary cells according to the AML differentiation state, ABCB1 activity and LSC compartment. Moreover, our results reveal the upregulation of ORAI1 and SOCE in chemoresistant ABCB1^high^ KG1 AML cells and in ABCB1^high^ AML primary cells after Ara-C exposure. However, Ara-C had the opposite effect in chemosensitive ABCB1^low^ U937 cells and ABCB1^low^ AML primary cells by inducing the downregulation of ORAI1 and SOCE. Several hypotheses can be formulated to explain these apparently contradictory results. First, the cell cycle engagement state of the two Rh123^low^ cell compartments from the KG1 and U937 cell lines is different. As previously mentioned, we observed a marked difference between the proportions of quiescent Ki67-negative cells, which were more pronounced in the U937 Rh123^low^ population, while the number of slow-cycling cells (Ki67low) was increased in KG1 Rh123^low^ cells. The difference in proportion in “true” quiescent G0 cells could explain the different behaviors in regard to SOCE modulation in response to Ara-C treatment. Moreover, the difference in basal ORAI1 expression levels between the two cell lines (significantly lower in KG1 than in U937 cells) may also explain the opposite response to Ara-C exposure regarding SOCE modulation. Nevertheless, further investigations are needed to better understand these differences between the two cell lines.

In a recent work, Borella et al. showed that lercanidipine, a CaV1.2 calcium channel inhibitor, combined with the chemotherapeutic agent Ara-C significantly decreased AML growth in a preclinical model, and that this effect was far more robust than when each molecule was applied separately [27]. These data emphasize the possible clinical value of calcium channel inhibition to increase AML chemosensitivity. However, based on our results, the basal level of ORAI1 channel expression, LSC size compartment, G0 quiescent state and ABCB1 activity could represent additional mechanisms of chemoresistance in AML that should also be considered when studying this issue.

The data presented herein clearly demonstrate that ORAI1 is expressed in both AML cell lines and in primary cells isolated from AML patients. We have shown the overexpression of ORAI1 in these leukemic cells compared with normal primary cells (PBMCs) isolated from healthy patients. These results suggest that AML cells, through ORAI1 upregulation, may acquire additional characteristics, such as apoptosis resistance. Previous studies have reported contradictory roles for SOCE and ORAI1 in apoptosis regulation. While it has been shown that SOCE and ORAI1 contribute to apoptosis induction by diverse stress-associated stimuli [6,28], other studies have demonstrated their prosurvival and antiapoptotic roles [29]. Our results in AML cells are, however, similar to our previous observations in pancreatic ductal adenocarcinoma (PDAC) cells, where ORAI1 also mediates SOCE and exhibits prosurvival and antiapoptotic roles in cell lines exposed to chemotherapy drugs. In this study on PDAC, we also demonstrated that drugs used in clinics (5-FU, gemcitabine) increase SOCE via the upregulation of ORAI1 and STIM1 [5]. Our data therefore show that in these two cancer models (i.e., AML and PDAC), the ORAI1 expression level is significantly higher than that in “normal” cells and is modified when cells are exposed to chemotherapy drugs.

It has been suggested that calcium channels display functional specificity in the activation of Ca^2+^-dependent transcription factors and the induction of gene expression. In this context, numerous studies have associated ORAI1-mediated Ca^2+^ entry with NFAT nuclear translocation and NFAT-dependent gene expression [30]. The precise role of NFAT in AML is still poorly documented. Nevertheless, it has been shown that the differentiation status of AMLs could impact NFAT expression levels [9]. A study on leukemic blasts isolated from AML patients was performed to determine the expression level (RNA-Seq) of NFAT isoforms according to differentiation status (FAB classification). NFATc2/c3 isoforms were found to be overexpressed at the early stage of differentiation. Moreover, it has been shown that NFAT inhibition increases the sensitivity to chemotherapy of primary AML leukemic blasts with an FL3-ITD mutation [11]. Interestingly, NFAT overexpression was observed in AML primary cells from patients during relapse compared with samples at the diagnostic stage [9]. While these data point to a possible role of NFAT in AML chemoresistance and AML progression, a majority of these studies only described *NFAT* gene expression without examining NFAT protein levels and intracellular distribution. In our study, ABCB1^high-^ and LSC-enriched chemoresistant cells (KG1 and AML#1 cells) displayed a preferential cytoplasmic localization of NFAT, while the ABCB1^low^ and LSC-enriched chemosensitive cells (U937 and AML#2 cells) presented a mostly nuclear distribution. Interestingly, Ara-C exposure induced NFAT translocation to the nucleus in ABCB1^high^ cells and cytoplasmic redistribution in ABCB1^low^ cells. These observations are in perfect agreement with the effect of Ara-C on SOCE in these cells, thus strongly suggesting a close functional link between SOC activity and NFAT regulation in AML cells.

There is growing evidence that NFAT signaling cooperates with mutations of the Fms-related tyrosine kinase receptor 3 (FLT3) receptor in AML. Internal tandem duplication of the FLT3 receptor (FLT3ITD) is present in approximately 25% of AML cases and confers particularly poor outcomes for patients compared with other AML subtypes [31]. While NFAT was found to negatively regulate genes that control cell cycle entry, such as Cdk4 and Cdk6, in normal myeloid cells, this activity was dependent on Flt3 ligand (Flt3-L) signaling and phospholipase PLCγ1-dependent calcium influx. Understanding the relationship between FLT3-L and NFAT activity could therefore be of utmost interest within the context of leukemia. Further investigations are thus required to better characterize the contribution of NFAT to the proliferation and quiescence of leukemic cells and LSCs and its possible role in chemoresistance.

In summary, our study presents for the first time the contribution of ORAI1, SOCE and the associated NFAT transcription factor in human AML cell lines and primary cells according to ABCB1 activity, LSC compartment and Ara-C chemoresistance. Further studies are needed to better understand the precise contribution of the ORAI1/SOCE/NFAT axis to LSC/leukemic cell cycle engagement and the specific mechanisms of apoptosis regulation by SOCE and ORAI1 to potentially reveal novel strategies targeting these proteins and improving current AML treatment efficiency.

## 4. Materials and Methods

**Cell culture.** KG1 and U937 cell lines were purchased from the ATCC^®^ (CCL-246™ and CRL-1593.2™, respectively). These cell lines were cultured at 37 °C in a humidified atmosphere with 5% CO_2_ in RPMI 1640 medium (Gibco, Waltham, MA, USA) supplemented with 1% penicillin streptomycin antibiotic cocktail (Gibco) and fetal bovine serum (FBS) (Gibco) at different concentrations according to the cell line needs (20 and 10% FBS for the KG1 and U937 cell lines, respectively). Cells were treated at the indicated concentrations with Ara-C (provided by Lille Hospital) or Synta66 (Sigma–Aldrich, Burlington, MA, USA).

**Patient samples.** Healthy and AML patient blood samples were processed as previously described [32] to collect peripheral blood mononuclear cells (PBMCs). Briefly, blood was first diluted in PBS (Gibco) and then gently added to human Pancoll solution (Pan Biotech, Aidenbach, Germany). After centrifugation, PBMCs were collected and used for experiments or frozen in 90% SVF and 10% DMSO solution in liquid nitrogen until use. After thawing, the cells were treated with DNase (Qiagen, Germantown, MD, USA) at a final concentration of 50 µg/mL and in RPMI supplemented with 10% FBS and 1% antibiotic cocktail.

**Rhodamine 123 (Rh123) exclusion assay, ABCB1 activity.** The Rh123 exclusion assay was performed as previously described [19]. Briefly, cells were adjusted to a concentration of 10^6^ per milliliter and loaded with 0.1 μg/mL Rh123 (Sigma–Aldrich, 𝜆ex 488 nm and 𝜆em 531 nm) in RPMI 1640 medium for 20 min at 37 °C in the dark for probe inclusion. After removing Rh123 from the medium, an exclusion step was performed at 37 °C for an additional 60 min. A positive control (cells with maximal Rh123 intensity) was obtained by keeping cells on ice or treating them with 0.1 µM verapamil (Sigma) to prevent Rh123 exclusion. Debris and cell doublets were excluded from the analysis, and cells were gated according to the positive control presenting the highest fluorescence intensity level. Cells with fluorescence intensity equal to the positive control were considered Rh123^high^ (lowest ABCB1 activity). Cells with the lowest Rh123 intensity were considered Rh123^low^ (highest ABCB1 activity). Acquisition was performed on an LSR-Fortessa X20 flow cytometer (BD Biosciences, Haryana, India).

**Calcium influx assay by flow cytometry.** Cells were loaded with the ratiometric dye indo-1-AM (Thermo Fisher Scientific, Waltham, MA, USA) as a Ca^2+^ indicator. One million cells per milliliter were loaded with 0.7 μM indo-1-AM in the corresponding medium for 30 min at 37 °C in the dark and then washed. The evolution of the intracellular calcium concentration ([Ca^2+^]_i_) was measured every 5 s as the F400 nm/F475 nm (RF400/F475) ratio of fluorescence with a flow cytometer UV light. Baseline [Ca^2+^]_i_ was acquired in a 0 mM Ca^2+^ solution containing 140 mM NaCl, 5 mM KCl, 1 mM MgCl_2_, 2 mM CaCl_2_, 5 mM glucose, and 10 mM HEPES (pH 7.4). After 120 s, the cells were treated with 1 µM of the sarco/endoplasmic reticulum calcium ATPase (SERCA) inhibitor thapsigargin (Focus Biomolecules). After 420 s of treatment with thapsigargin, a Ca^2+^-containing solution was added to the cells (final [Ca^2+^]e 2 mM). Fluorescence was monitored with an LSR-Fortessa X20 flow cytometer, and median fluorescence values (*n* = 1000–10,000 cells for each time point) were extracted for further analysis. The Ca^2+^ release (ΔrRF400/F475) and capacitative calcium entry (also noted SOCE for store-operated calcium entry) (ΔiRF400/F475) were calculated as previously described [20]. Graphs were plotted using GraphPad Prism software.

**Immunophenotyping.** After rinsing, the cells were stained with fluorochrome-conjugated antibodies (all from Biolegend, San Diego, CA, USA) for 30 min at room temperature (RT) in PBS with 10% SVF. Antibodies were used at a 1:100 dilution (except for the PE-Cy7-conjugated CD33 antibody used at a 1.25:1000 dilution), and matching isotype antibodies were used at the same final concentration. Then, the cells were rinsed and resuspended in PBS before being analyzed by flow cytometry. Compensation beads (Invitrogen) were used to establish a matrix of compensation. Gating was determined based on negative control cell staining with corresponding isotype antibodies. Depending on the protocol, two panels of antibodies were used. If combined with Rh123 exclusion and calcium influx assays, allophycocyanin (APC)-conjugated CD34 and phycoerythrin (PE)-conjugated CD38 antibodies were used. If combined with the calcium influx and cell viability assays, APC-conjugated CD34, PE-conjugated TIM3, PE-Cy7-conjugated CD33 and Brilliant Violet 421 (BV421)-conjugated CD38 antibodies were used. To assess their viability, cells were loaded with a DyeTM 750/777 Fixable Viability Staining Kit for 30 min at RT and protected from light.

**Proliferation assay.** Proliferation status was determined by nuclear Ki67 protein staining with a fluorescein isothiocyanate (FITC)-conjugated Ki67 antibody (Abcam, Cambridge, UK). Cells were first fixed with a 4% paraformaldehyde solution (Sigma–Aldrich) for 10 min at RT and then washed and incubated for 30 min at RT with 0.1% Triton X100 (Sigma–Aldrich) and 10% SVF solution supplemented with PBS. After washing, the cells were incubated with APC-conjugated CD34, BV421-conjugated CD38, PE-Cy7-conjugated CD33, PE-conjugated TIM3 antibodies (all from Biolegend) and FITC-conjugated Ki67 antibody (Abcam) in 0.1% Triton X100 and 10% SVF solution in PBS for 30 min at 4 °C in the dark. Cells were stained with the antibodies used at a 1:100 dilution (except for the PE-Cy7-conjugated CD33 used at a 1.25:100 dilution) or with matching isotypes at the same final concentration. Cells were then placed on ice before flow cytometry analysis.

**Cell cycle analysis.** Cells were adjusted to a concentration of 10^6^ per milliliter to be fixed and permeabilized with 70% ethanol (Sigma) for 30 min at −20 °C. Cells were stained for 30 min at RT in the dark with 50 µg/mL propidium iodide (Sigma–Aldrich) and 5 µg/mL RNase (Qiagen) in PBS. To determine the percentage of cells in the G0 phase of the cell cycle, cells were preincubated with the FITC-conjugated Ki67 antibody (Abcam) at a 1:100 dilution or with matching isotype at the same final concentration in PBS with 10% SVF for 30 min at 4 °C in the dark. Then, the cells were kept on ice until analysis by flow cytometry.

**Cell sorting.** Subsequently, for the Rh123 exclusion assay, U937 and KG1 cells were washed and resuspended to a concentration of 5 × 10^6^ cells per milliliter in culture cell medium without phenol red and 1% SVF (Sigma–Aldrich). Rh123^low^ and Rh123^high^ cells were sorted on a FACSAria flow cytometer (BD Biosciences). Gating was determined based on the positive control loaded with the Rh123 probe without the exclusion step. After sorting, the cells were kept at 37 °C before being used in further experiments.

**Flow cytometry data analysis**. Data were analyzed using FlowJo software (TreeStar). Live cells were discriminated from dead cells using a viability probe (DyeTM 750/777). Cell subpopulations were discriminated according to their rhodamine exclusion and/or cell-surface marker expression. Calcium mobilization was analyzed in the different gated subpopulations.

**Immunocytochemistry.** Cells were immobilized on 0.01% poly-lysine (Sigma–Aldrich)-coated coverslips at a concentration of 10^6^ cells per coverslip for 10 min (or 24 h if treated) at 37 °C before being fixed with a 4% PAF solution (Sigma–Aldrich) for 10 min at RT. After washing, the cells were treated with a 0.1% Triton X-100 saturation/permeabilization solution for 10 min at RT. PBS-Tween (0.1% of Tween in PBS, Merck) was used to wash the cells for 5 min before incubating them in blocking buffer (1% BSA in PBS, Sigma–Aldrich) for 60 min at RT. Primary polyclonal rabbit anti-human NFAT antibody (1:100 dilution, Abcam) was directly added to the blocking buffer for 60 min at RT. After washing with PBS-T, FITC-conjugated Ki67 (1:100 dilution, Abcam) and Alexa Fluor 568 donkey anti-rabbit secondary antibody (1:2000 dilution, Invitrogen, Waltham, MA, USA) were subsequently added for another 60 min at RT. Negative controls were performed by replacing the primary antibody and fluorochrome-conjugated antibody with irrelevant antibodies of the same isotype. To visualize nuclei, 10 μg/mL Hoechst (Molecular Probes, Eugene, OR, USA) was added for 10 min at RT. The coverslips were mounted on microscope slides with Fluoroshield mounting medium (Abcam). For confocal microscopy, cell images were obtained using a confocal laser scanning microscope (LSM 700, Carl Zeiss MicroImaging GmbH, Jena, Germany) with a Plan Apochromat 40×/1.3 numerical aperture oil immersion objective (Hoechst, Frankfurt, Germany, 𝜆ex 343 nm, 𝜆em 483 nm, NFAT 𝜆ex 568 nm, 𝜆em 605 nm and Ki67 𝜆ex 488 nm, 𝜆em 532 nm). Images were analyzed with Zeiss LSM Image Browser software.

**siRNA-mediated gene knockdown.** To evaluate the role of the ORAI1 calcium channel in SOCE, five million KG1 or U937 cells were transfected by electroporation in transfection buffer (LONZA Amaxa^®^ Cell Line Nucleofactor™ Kit V, Basel, Switzerland) with 100 nM control or ORAI1-specific siRNA (Santa Cruz Biotechnology, Dallas, TX, USA) to downregulate ORAI1 expression. After passing the cells through the Amaxa Nucleofactor™ II electroporator, they were cultured in 6-well plates. Forty-eight hours post-transfection, the cells were collected, and *ORAI1* expression was assessed by qRT–PCR. The gene silencing efficiency was approximately 90% and 95% for KG1 and U937 cell lines, respectively (Figure S9).

**RNA Extraction, qRT–PCR.** RNA extraction was performed following the manufacturer’s protocol (Qiagen RNeasy Mini Kit). RNA was transcribed into cDNA using random hexamers and the High Capacity Reverse Transcription kit from Applied Biosystems (Foster City, CA, USA). All qRT-PCRs were performed using TaqMan fluorescent probes (human *ORAI1, MMRN1, LAPTM4B, NYNRIN, KLF4, SOX2, NANOG, DNMT3B, ABCB1* and *BETA-2 MICROGLOBULIN* or *B2M*) provided by Applied Biosystems, Bedford, MA, USA. Duplex qPCR was performed, and discrimination of the genes of interest from the reference gene was allowed owing to 2 distinct fluorophore dyes, FAM and VIC probes for the genes of interest and the reference gene, respectively. The relative expression ratio for each gene was calculated by the 2^−ΔΔCt^ method. The calculated values represent the expression level for each gene relative to the expression of *B2M*, which was used as the endogenous control. No expression denotes no detection in 50 ng of cDNA.

**Analysis of public datasets**. RNA-seq datasets of human AML samples at diagnosis (*n* = 439) [12] are available in the public domain (https://www.cbioportal.org, accessed on 15 March 2022). The RNA-seq expression unit used was counts per million reads mapped (CPM). Raw data were extracted and analyzed as previously described [13,14].

**Statistical analyses.** All results are expressed as the means  ±  SEM of at least 3 independent experiments with 3 replicates each. For public dataset analysis, the number of patient samples is indicated. Comparisons between means were assessed using the Student’s t test for unpaired data or one-way ANOVA. For the Student’s t test, if unequal variance was observed, then Welch’s correction was applied. Statistical analyses were performed using GraphPad Prism software. A *p* value  ≤ 0.05 was considered significant. Asterisks denote * *p* < 0.05, ** *p* < 0.01 and *** *p* < 0.001. The leukemic stem cell (LSC) score was determined as described previously [15].

## Figures and Tables

**Table 1:** Relative expression of LSC genes, *ABCB1* and LSC compartment proportion in KG1 and U937 AML cell lines. RT–qPCR was performed to analyze the relative expression of the indicated genes as described in the Materials and Methods section. The percentage of LSCs (CD34 + CD38−) was also analyzed by flow cytometry. * *p* < 0.05; *** *p* < 0.001; **** *p* < 0.0001.

AML Cell Lines	«LSC» Gene Expression				MDR Gene Expression	Percent of «LSC»
	*LAPTMB4*	*NYNRIN*	*MMRN1*	*DNMT3B*	*ABCB1*	CD34+/CD38−
KG1	1	1	1	1	1	10.4 ± 0.8 ***
U937	Not expressed	Not expressed	0.05 ± 0.01 ***	0.37 ± 0.3 *	0.001± 0.0002 ****	0.04 ± 0.01

**Table 2:** LSC compartment identified by CD34 + CD38− phenotype combined with the Rh123 exclusion assay in KG1 and U937 AML cell lines. Percentage of Rh123^low^ and Rh123^high^ cell compartments associated with or without CD34 and CD38 surface markers analyzed by flow cytometry. Means were compared between KG1 and U937 cells. ** *p* ≤ 0.01.

AML Cell Lines	Rh123^high^	Rh123^low^	CD34+/CD38−	Rh123^high^ CD34+/CD38−	Rh123^low^ CD34+/CD38−
KG1	0.09 ± 0.05 **	99.9 ± 0.05 **	10.4 ± 0.8 **	0.0025 ± 0.02	10.4 ± 0.8 **
U937	97.1 ± 0.8	2.9 ± 0.8	0.04 ± 0.01	0.02 ± 0.003	0.02 ± 0.003

## Data Availability

Data are available in a publicly accessible repository that does not issue DOIs. Publicly available datasets were analyzed in this study. These data can be found here: https://www.cbioportal.org, accessed on 15 March 2022.

## Supplementary Materials

The following supporting information can be downloaded: https://www.mdpi.com/1422-0067/23/10/5555/s1.
