# Supplementary material for: Involvement of ORAI1/SOCE in Human AML Cell Lines and Primary Cells According to ABCB1 Activity, LSC Compartment and Potential Resistance to Ara-C Exposure
Source: Int J Mol Sci. 2022 May 16;23(10):5555. doi: 10.3390/ijms23105555 (PMC9141756; doi:10.3390/ijms23105555)
Supplement: Supplementary file 1 [file ijms-23-05555-s001.zip › ijms-1707132-SI.pdf]

Table S1. Patient characteristics including age, sex, the French-American-British (FAB) classification of AML, the European LeukemiaNet (ELN) risk classification: favorable, intermediate or adverse risk, the percent of CD34+CD38- cells and Leukemic Stem Cell (LSC) score; N.D.: No Data

**Patient**	**Age (y)**	**Sex**	**FAB**	**ELN**	**CD34+CD38- cells**	**LSC score***
AML#1	34	M	M0	3- adverse	82%	ND
AML#2	48	F	M5	ND	0.3%	ND
AML#3	30	M	M4	ND	18.4%	ND
AML#4	57	M	ND	ND	0.01%	ND
AML#5	72	F	M2	Favorable	18.2%	1
AML#6	78	M	M0	3- adverse	46%	3 inter II
AML#7	69	M	M2	3- adverse	22.0%	3 inter II
AML#8	50	F	M2	Favorable	<0.01%	ND
AML#9	69	M	M2	2- inter	ND	2 inter I

Table S2. Differential expression patterns of the *17 LSC gene signature* described by Ng et.al [15] in ABCB1^{high} and ABCB1^{low} AML patients subgroups. RNA-seq data were generated by Tyner et al. [12] from n= 439 samples derived from patients with AML at diagnosis of the disease. P-value and q-value were indicated according to Student t-test and Benjamini Hochberg procedure respectively. Bold gene names are genes with significant differences.

(17 LSC gene signature) LSC genes[1]	ABCB1^{high}	ABCB1^{low}	p- value (derived from Student t-test)	q-value (derived from Benjamini Hochberg procedure)
CD34	**higher expression**	**lower expression**	**1.09^{e}-21**	**3.97^{e}-19**
LAPTM4B	**higher expression**	**lower expression**	**1.808^{e}-4**	**1.459^{e}-3**
NYRMIN	**higher expression**	**lower expression**	**0. 00529**	**0.0865**
MMRNI	**higher expression**	**lower expression**	**1.97^{e}-8**	**2.03^{e}-7**
DNMT3B	**higher expression**	**lower expression**	**2.521^{e}-4**	**8.279^{e}-4**
KIAA0125	ND	ND	-	-
SOCS2	higher expression	lower expression	0.463	0.542
CPXM1	higher expression	lower expression	0.0923	0.139
AKR1C3	**higher expression**	**lower expression**	**1.0310^{e}-9**	**1.52e-8**
GPR56	ND	ND	-	-
CDK4	**higher expression**	**lower expression**	**0.0210**	**0.0390**
CDK6	higher expression	lower expression	0.163	0.225
DPYSL3	lower expression	higher expression	0.0923	0.139
NGFRAP1	higher expression	lower expression	ND	ND
ARHGAP22	higher expression	lower expression	0.869	0.900
LOC284422	ND	ND	-	-
ZBTB46	**higher expression**	**lower expression**	**3.69^{e}-9**	**4.69^{e}-8**

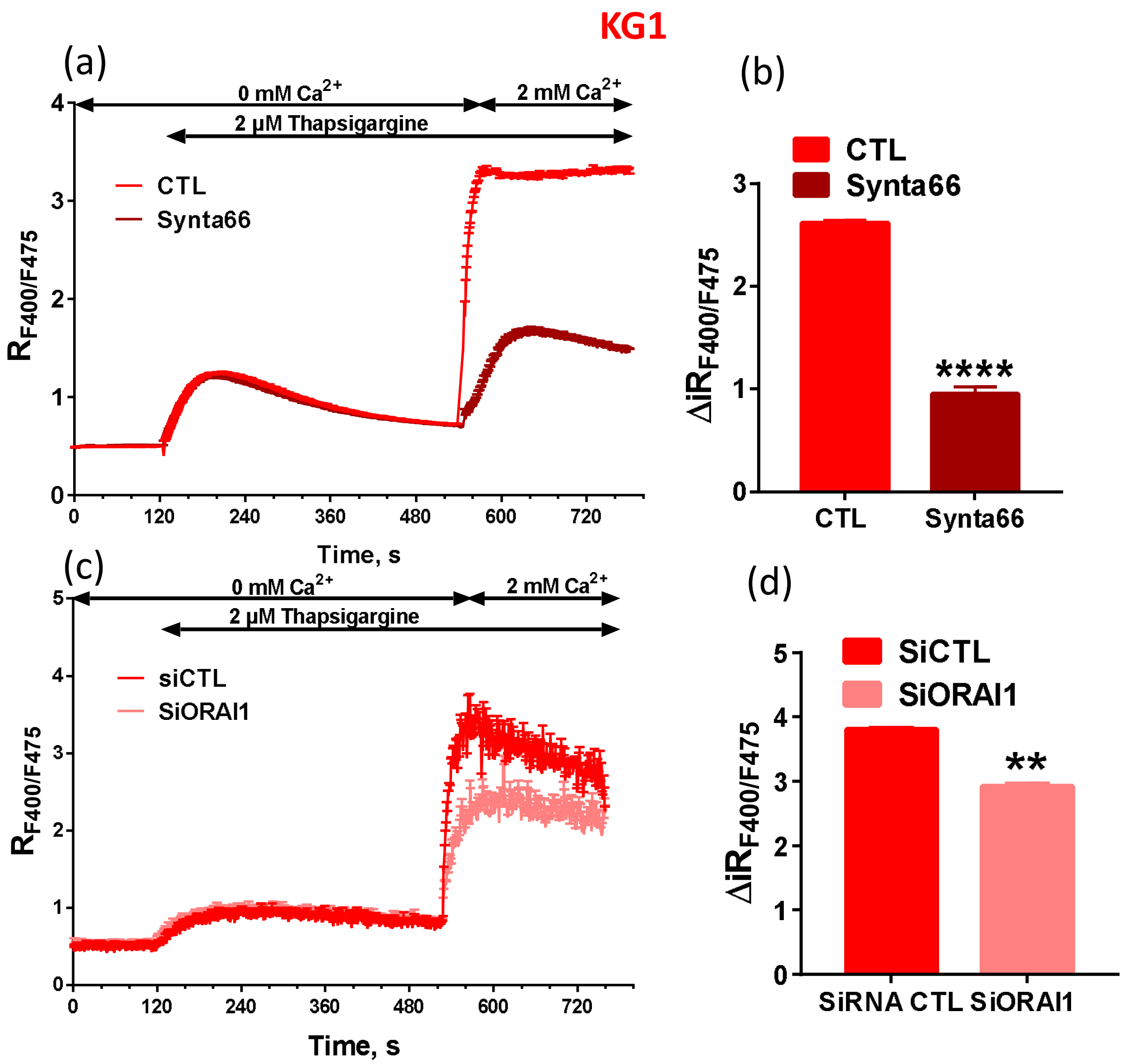

Figure S1. ORAI1 is involved in SOCE in KG1 leukemic cell line. Time course of Ca^{2+} cytosolic concentration using the Indo-AM ratiometric dye in KG1 cell line treated with the SOC channel inhibitor agent synta66 (a) or transfected with the control siRNA and siORAI1 (c). Each point represent mean of 3 independent experiments regrouping between 1,000 -10,000 cells. Calcium capacitive entry of KG1 leukemic cell line treated with synta66 (b) or transfected with the control siRNA and siORAI1 (d). ** $p \leq 0.01$, ****$p \leq 0.0001$ .Three independent experiments were performed.

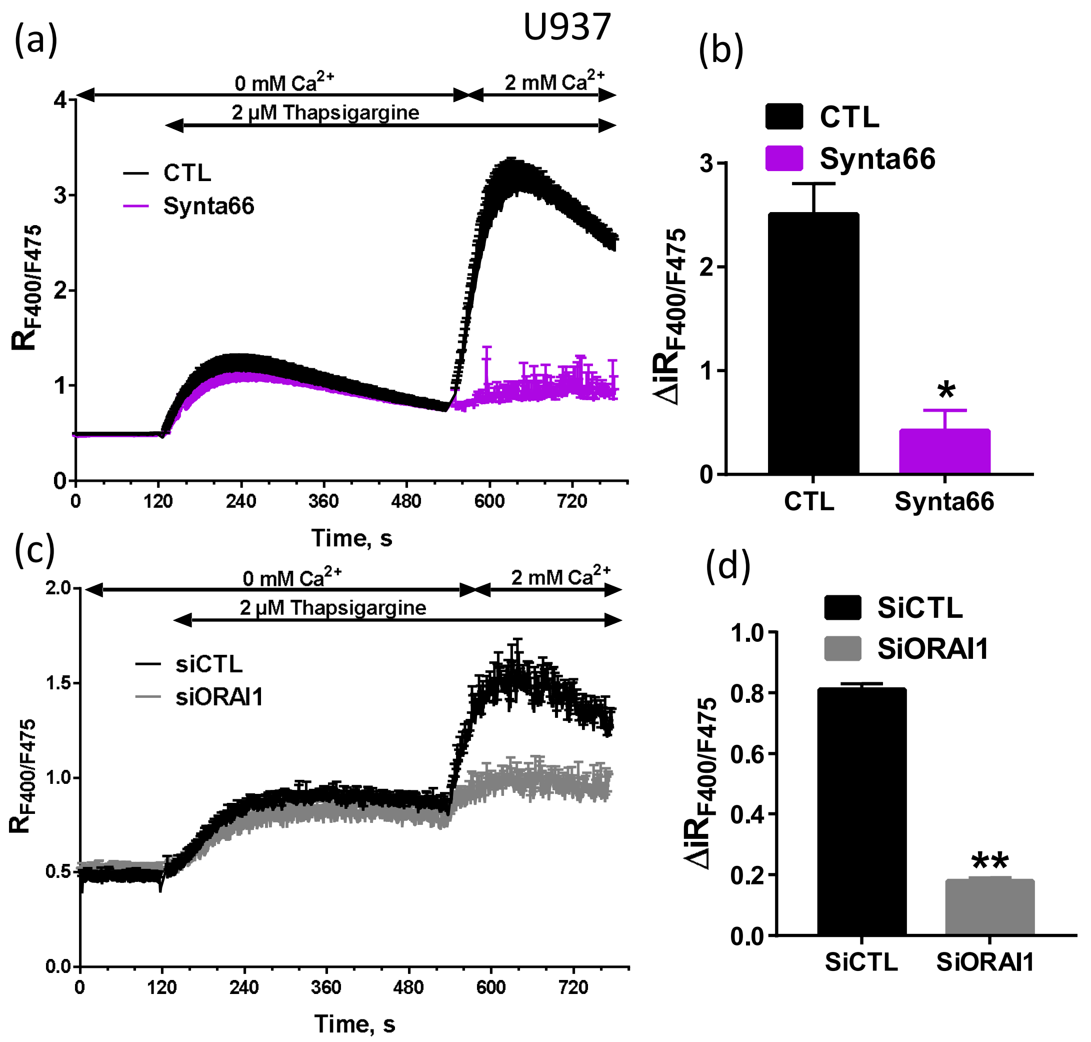

Figure S2. ORAI1 is involved in SOCE in U937 leukemic cell line. Time course of Ca^{2+} cytosolic concentration using the Indo-AM ratiometric dye in KG1 cell line treated with the SOC channel inhibitor agent synta66 (a) or transfected with the control siRNA and siORAI1 (c). Each point represent mean of 3 independent experiments regrouping between 1,000 -10,000 cells. Calcium capacitive entry of U937 leukemic cell line treated with synta66 (b) or transfected with the control siRNA and siORAI1 (d). * $p \leq 0.05$), **$p \leq 0.01$ Three independent experiments were performed.

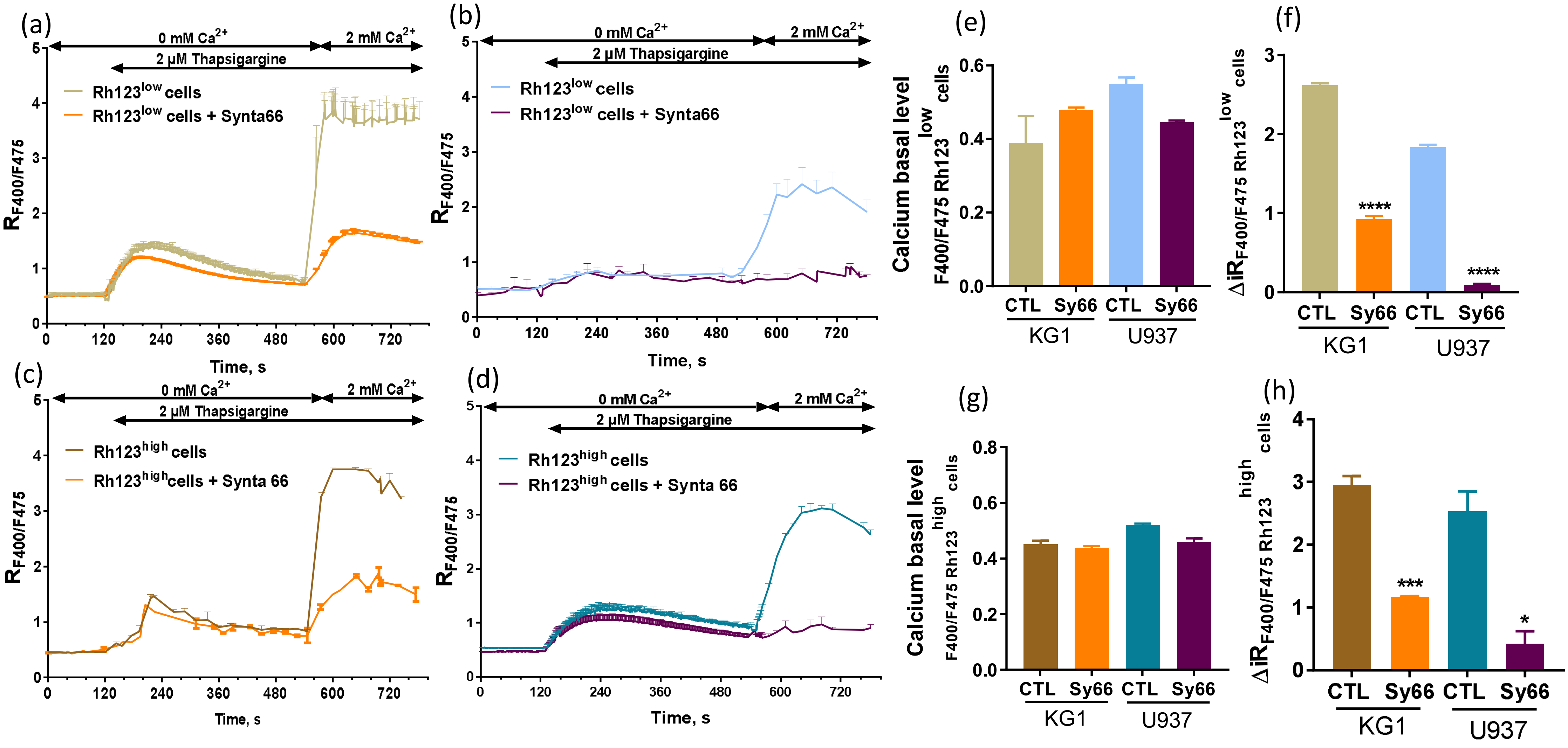

Figure S3. SOCE in Rh123^{low} and Rh123^{high} populations in KG1 and U937 cell lines. Cells were treated or not with 10 µM synta66. Time course of Ca^{2+} cytosolic concentration using the Indo-AM ratiometric dye in the KG1 (a and c) and U937 (b and d) Rh123^{low} and Rh123^{high} populations respectively. Each point represent mean of 3 independent experiments regrouping between 1,000 -10,000 cells. Analysis by flow cytometry of the F400/F475 ratio reflecting the calcium basal level and the capacitive Ca^{2+} entry in the KG1 and U937 Rh123^{low} (e and f respectively) and Rh123^{high} (g and h respectively) subpopulations. * $p \leq 0.05$, *** $p \leq 0.001$, **** $p \leq 0.0001$. Three independent experiments were performed.

Table S3. LSC compartment identified by CD34+CD38- phenotype combined with Rh123 exclusion assay in KG1 and U937 AML cell lines. Percentage of $Rh123^{low}$ and $Rh123^{high}$ cell compartment associated with or without CD34 and CD38 surface markers analyzed by flow cytometry in control condition , after 24 h of treatment with synta66 10µM , transfected cells with control SiRNA or SiORAI1.

AML cell lines		$Rh123^{high}$	$Rh123^{low}$	$CD34^{+}$ $CD38^{-}$	$Rh123^{high}$ $CD34^{+}$ $CD38^{-}$	$Rh123^{low}$ $CD34^{+}$ $CD38^{-}$
**KG1**	**Control**	0.084±0.031	99.93±0.033	13.23±3.6	0.002±0.002	13.2±3.6
	**Synta66**	0.083±0.026	99.93±0.033	14.2±5.2	0.0022±0.01	14.2±5.2
**U937**	**Control**	96.83±0.85	3.14±0.85	0.04±0.011	0.013±0.01	0.03±0.01
	**Synta66**	95.5±2	4.5±2.1	0.03±0.01	0.01±0.001	0.02±0.01
**KG1**	**SiCTL**	0.092±0.012	99.32±0.01	15.25±3.3	0.0018±0.002	15.25±3.3
	**SiORAI1**	0.098±0.032	99.28±0.02	16.2±2.2	0.0032±0.02	16.2±2.2
**U937**	**SiCTL**	95.84±0.65	4.15±0.25	0.05±0.01	0.022±0.01	0.04±0.01
	**SiORAI1**	93.2±2	6.5±2.2	0.04±0.01	0.02±0.001	0.03±0.01

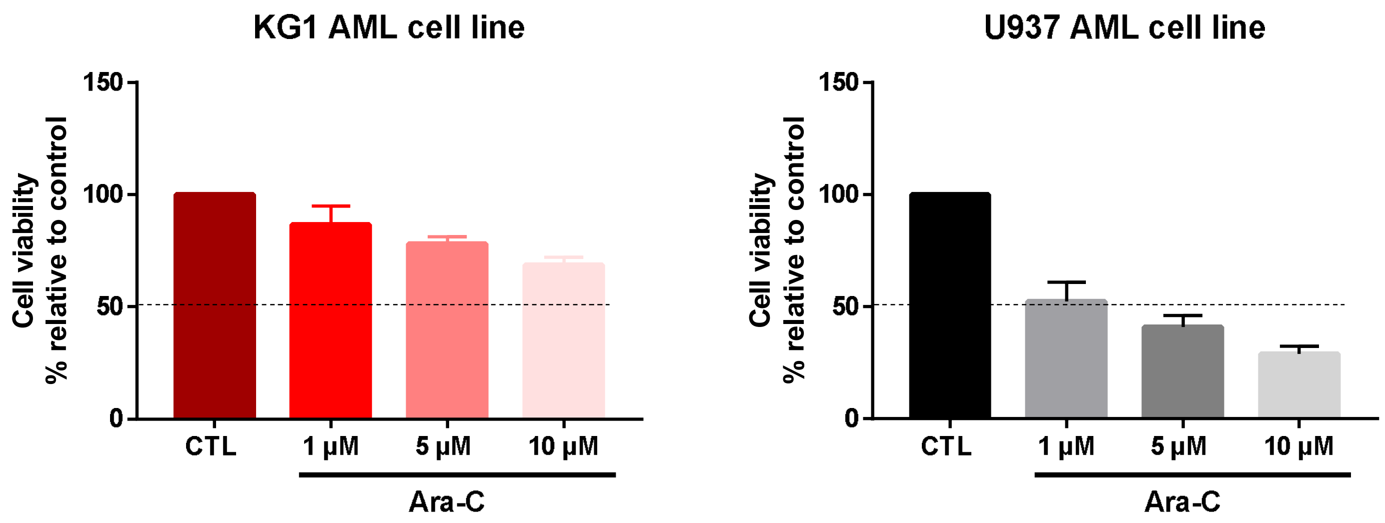

Figure S4. KG1 and U937 cell viability following Ara-C treatment. Viable cells were counted by exclusion trypan blue after a Ara-C exposure at indicated concentrations during 24 hours .

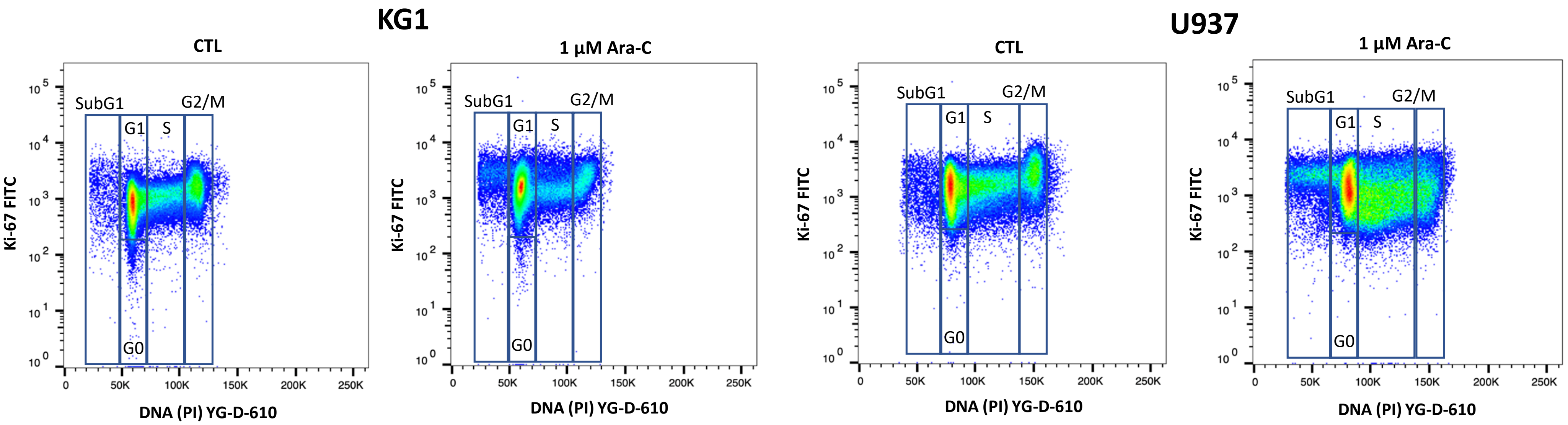

Figure S5. Cell cycle analysis of KG1 and U937 AML cell lines after Ara-C treatment. Cells were treated for 24 hours with 1 Ara-C during 24h and cell cycle analysis were assessed following Ki-67 and Propidium Iodide (PI) double staining.

Table S5. LSC compartment identified by CD34+CD38- phenotype combined with Rh123 exclusion assay in KG1 and U937 AML cell lines after Ara-C treatment. Percentage of Rh123^{low} and Rh123^{high} cell compartment associated with or without CD34 and CD38 surface markers analyzed by flow cytometry, in control condition or after 24 h of treatment with the indicated concentration of Ara-C. * $p \leq 0.05$

AML cell lines		Rh123^{high}	Rh123^{low}	CD34^{+} CD38^{-}	Rh123^{high} CD34^{+} CD38^{-}	Rh123^{low} CD34^{+} CD38^{-}
KG1	**Control**	0.036±0.006	99.93±0.03	4.8±0.87	0.01±0.002	4.8±0.9
	**Ara-C 1 μM**	0.05±0.016	99.93±0.03	8.5±0.5*	0.02±0.01	8.5±0.5*
	**Ara-C 5 μM**	0.040±0.009	99.97±0.03	9.8±0.5*	0.01±0.003	9.8±0.5*
	**Ara-C 10 μM**	0.057±0.010	99.97±0.03	9.95±0.08*	0.02±0.003	9.94±0.1*
U937	**Control**	97.1±0.794	2.9±0.8	0.04±0.01	0.02±0.003	0.02±0.003
	**Ara-C 1 μM**	80.83±7.1	19.2±7.1	0.04±0.012	0.01±0.002	0.034±0.01*
	**Ara-C 5 μM**	67.133±5	32.9±5	0.063±0.02	0.013±0.003	0.05±0.02*
	**Ara-C 10 μM**	68.4±3.55	31.6±3.6	0.06±0.02	0.014±0.003	0.05±0.02*

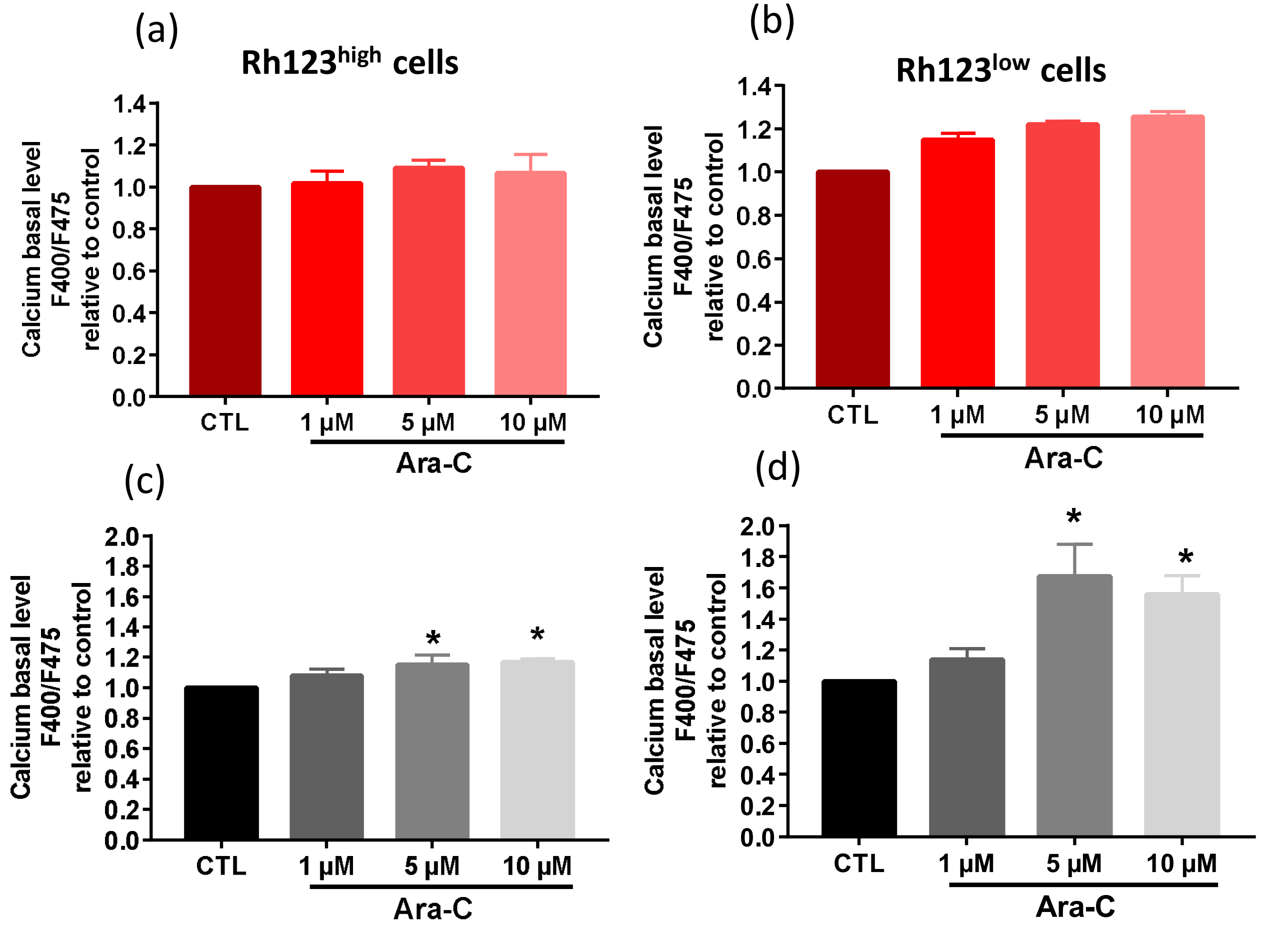

Figure S6. Analysis by flow cytometry of the F400/F475 ratio reflecting the calcium basal level in the KG1 and U937 Rh123^{high} (a and c respectively) and Rh123^{low} (b and d respectively) populations treated or not with the indicated concentrations of Ara-C. * $p \leq 0.05$

Table S6. LSC compartment identified by CD34+CD38- phenotype combined with Rh123 exclusion assay in AML#1 and AML#2 primary cells after Ara-C treatment. Percentage of $Rh123^{low}$ and $Rh123^{high}$ cell compartment associated with or without CD34 and CD38 surface markers analyzed by flow cytometry in control condition or after 24 h of treatment with indicated concentration of Ara-C.

**AML Patient #1**	**$Rh123^{high}$**	**$Rh123^{low}$**	**$CD34^{+}$ $CD38^{-}$**	**$Rh123^{high}$ $CD34^{+}$ $CD38^{-}$**	**$Rh123^{low}$ $CD34^{+}$ $CD38^{-}$**
**Control**	2	98	82	0.009	81.9
**Ara-C 5μM**	2	98	78	0.047	78
**AML Patient #2**	**$Rh123^{high}$**	**$Rh123^{low}$**	**$CD34^{+}$ $CD38^{-}$**	**$Rh123^{high}$ $CD34^{+}$ $CD38^{-}$**	**$Rh123^{low}$ $CD34^{+}$ $CD38^{-}$**
**Control**	58.8	40.2	0.3	0.1	0.2
**Ara-C 5μM**	25.7	74.3	0.3	0.06	0.3

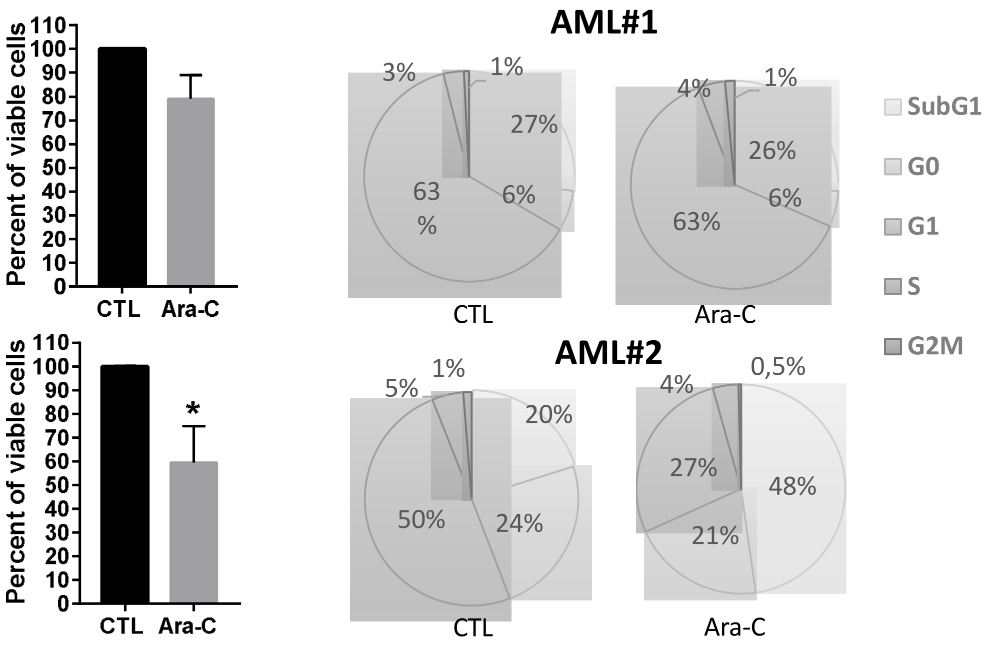

Figure S7. Effect of Ara-C on viability and cell cycle in AML patient #1 and patient #2 . Cells were either in a control condition or treated for 24h with Ara-C 5 µM. Two independent experiments were performed. Percentage of viable cells counted with trypan blue exclusion staining. Cell cycle analysis with Ki67/PI staining of AML#1 and AML#2 in control condition and following 5 µM Ara-C treatment.

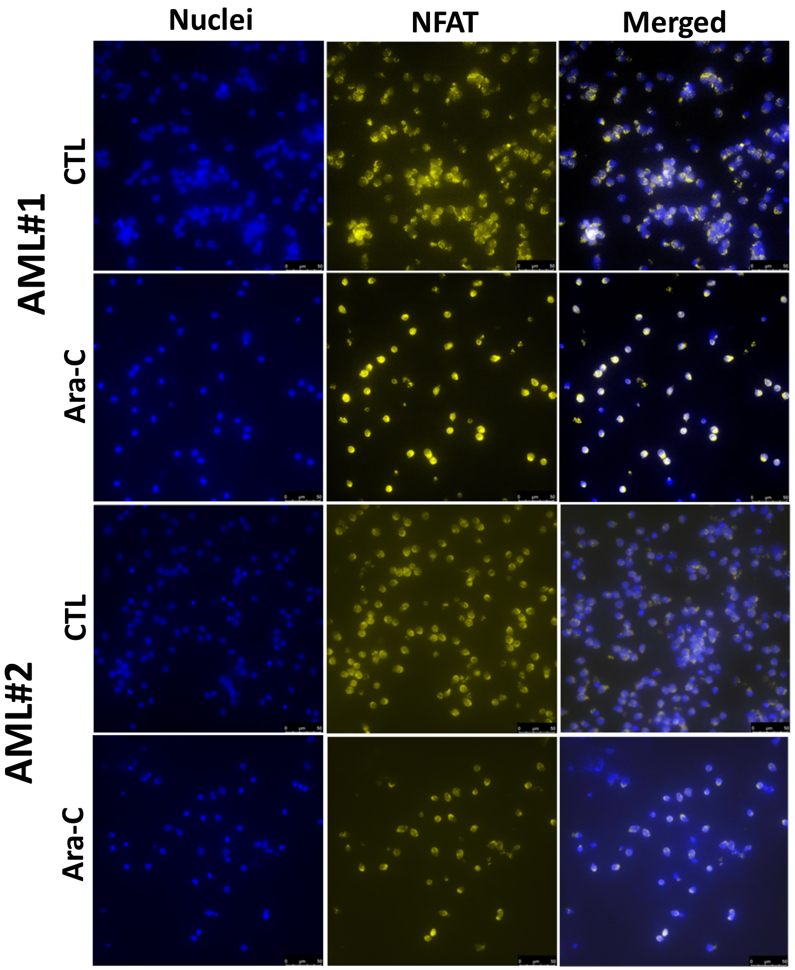

Figure S8. Effect of Ara-C on the NFAT localization in AML primary cells. Confocal analysis showing expression and localization of NFAT (Alexa Fluor 568) and nuclei (Hoechst, blue) in control and Ara-C-treated cells conditions in the AML patient #1 and #2.

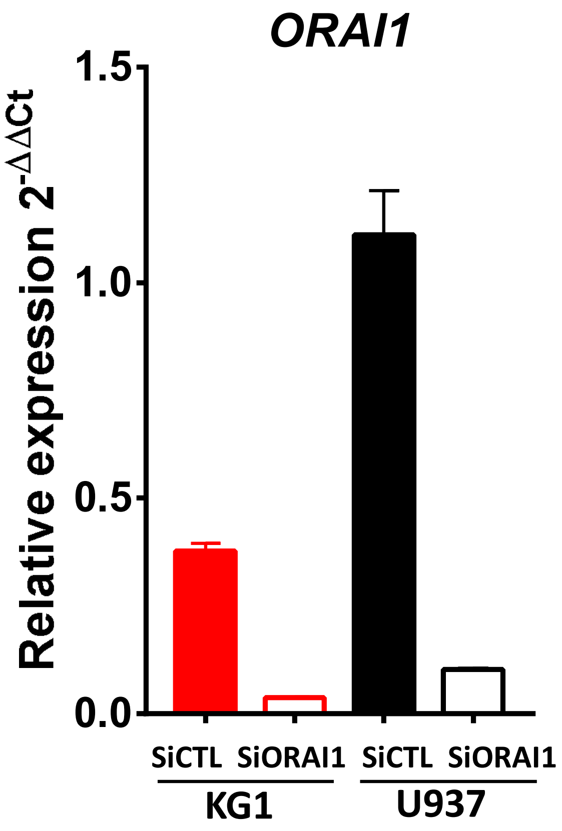

Figure S9. Relative ORAI1 expression in transfected KG1 and U937 cells with control SiRNA (SiCTL) and ORAI1 SiRNA (SiORA1) determined by RT-qPCR.
